# Water-mediated recycling of gold, palladium and platinum using semimetallic TiS_2_ and TaS_2_ nanosheets

**DOI:** 10.1093/nsr/nwaf522

**Published:** 2025-11-20

**Authors:** Jianhong Wei, Miaofei Huang, Kuang Yu, Huanjing Liang, Fei Li, Kaiqiang Zheng, Fangluo Chen, Yibo Gao, Yang Su, Hui-Ming Cheng

**Affiliations:** Institute of Materials Research, Shenzhen International Graduate School, Tsinghua University, Shenzhen 518055, China; Institute of Materials Research, Shenzhen International Graduate School, Tsinghua University, Shenzhen 518055, China; Institute of Materials Research, Shenzhen International Graduate School, Tsinghua University, Shenzhen 518055, China; Institute of Materials Research, Shenzhen International Graduate School, Tsinghua University, Shenzhen 518055, China; Institute of Materials Research, Shenzhen International Graduate School, Tsinghua University, Shenzhen 518055, China; Institute of Materials Research, Shenzhen International Graduate School, Tsinghua University, Shenzhen 518055, China; Institute of Materials Research, Shenzhen International Graduate School, Tsinghua University, Shenzhen 518055, China; Institute of Materials Research, Shenzhen International Graduate School, Tsinghua University, Shenzhen 518055, China; Institute of Materials Research, Shenzhen International Graduate School, Tsinghua University, Shenzhen 518055, China; Institute of Technology for Carbon Neutrality, Shenzhen Institutes of Advanced Technology, Chinese Academy of Sciences, Shenzhen 518055, China; Faculty of Materials Science and Energy Engineering, Shenzhen University of Advanced Technology, Shenzhen 518055, China; Shenyang National Laboratory for Materials Science, Institute of Metal Research, Chinese Academy of Sciences, Shenyang 110016, China

**Keywords:** precious-metal recycling, transition-metal dichalcogenides, two-dimensional materials, sustainability

## Abstract

The intensive and irreplaceable consumption of precious metals (PMs) including gold (Au), palladium (Pd) and platinum (Pt) in the electronic and catalysis industries, coupled with their scarcity in Earth’s crust, demand innovative recycling solutions for PM sustainability. However, efforts to recycle PMs from leachates of their waste are frustrated by an unsatisfactory extraction capacity at low concentrations and remain predominantly focused on gold, leaving other PMs largely unexplored. We report the ultrahigh reductive recycling of PM ions and their simultaneous aqueous-phase deposition on semimetallic transition-metal dichalcogenides of TiS_2_ and TaS_2_ nanosheets_._ Notably, TiS_2_ shows unprecedentedly high extraction capacities of ∼8, 2.3 and 1.15 g/g for Au, Pd and Pt ions, respectively, and the adsorbed PM ions are directly transformed into nanoparticles deposited on the nanosheets. Mechanistic studies reveal that water-mediated electron donation from the sulfur site of the semimetallic TiS_2_ and TaS_2_ nanosheets is responsible for the ultrahigh extraction capacity, with a single TiS_2_ molecule donating >13 electrons to gold ions. This electron transfer is mediated by the formation of sulfur-oxygen species during water dissociation. We further demonstrate the selective and complete recovery of Au, Pd and Pt from real-world waste streams including electronic waste, spent catalysts and automotive catalytic converters.

## Teaser

2D TiS_2_ and TaS_2_ nanosheets show ultrahigh adsorption capacities for low-concentration Au, Pd and Pt ions, allowing the efficient recovery of precious metals from their waste streams.

## INTRODUCTION

Precious metals (PMs), including gold (Au), platinum (Pt), palladium (Pd) and others, are the cornerstones of many industrial processes and devices, ranging from catalysis and renewable-energy apparatus to modern electronic appliances [1–6]. For instance, Au is essential in electronics, while Pt and Pd are crucial for industrial catalysis [7–12]. However, the high annual consumptions of ∼4600 tons of Au, ∼220 tons of Pt and ∼300 tons of Pd [13–15], their low concentrations in Earth’s crust (∼4, 5 and 13 mg/ton) [16] and their low recycling rates of <20% present sustainability challenges [14,15]. Recycling PMs from wastes offers a sustainable alternative to ore mining, as it reduces the energy consumption, resource extraction and environmental impact caused by the landfilling of such wastes [9,17].

Hydrometallurgy is frequently used for PM recycling and involves leaching to dissolve the PMs and coexisting elements, followed by the selective adsorption and reduction of the PM ions [13]. Selective adsorption of diluted PM ions from its leachate-containing complex composition is a critical step and relies on the efficiency of the adsorbents [12,18]. Traditional adsorbents have the limitations of unsatisfactory selectivity and insufficient extraction capacity (*Q_e_*) [19–22]. Recent advances show that, by incorporating redox sites (primarily amine groups) into porous materials, including porous polymers [23], metal–organic frameworks [24,25] and porous aromatic frameworks [26,27], each redox site, and also chemisorption site, donates one or two electrons and the resulting adsorbents have a *Q_e_* of 300–4000 mg/g (at an Au concentration of <200 ppm) and simultaneously reduce the ionic gold to the element. While gold adsorbents have been extensively studied, adsorbents for other PMs, such as Pt, are largely unexplored despite their high economic value and critical application in the catalysis industry; for example, existing adsorbents for Pt rely on either weak intermolecular forces [28,29] or use gold-specific adsorbents, which are efficient for gold recovery, but ineffective for other PMs [10,23,30,31], and they suffer from unsatisfactory adsorption performance for Pt ions because of its lower reduction potential than that of Au.

Recently emerging 2D materials, with their large surface area and important surface chemistry, present new opportunities for efficient PM recycling [32,33]. Our previous study has shown that reduced graphene oxide (rGO) is highly efficient for Au recycling, but is ineffective for [PtCl_6_]^2–^ due to its inability to reductively adsorb Pt ions [32]. Though other 2D materials are reported to reductively adsorb Pt ions, they also exhibit low *Q*_e_ even at a high concentration due to their weak electron-donation capability to Pt ions and they suffer from either a complicated fabrication process and/or a high cost, undermining their economic viability for the practical recycling of PMs [4,30,34,35].

Materials that enable efficient electron donation to PM ions should be promising adsorbents for PM recycling. We then became interested in 2D transition-metal dichalcogenides (TMDs)—in particular, semimetallic TMDs, such as TiS_2_ and TaS_2_, which have narrow bandgaps (∼0.2 and ∼0.1 eV) and high Fermi levels of approximately –4.13 and –4.49 eV [36–40], respectively, which are well above the equivalent energy levels for the reduction potentials of [AuCl_4_]^−^, [PdCl_4_]^2–^ and [PtCl_6_]^2–^ ions; during the adsorption process, their 2D feature allows the exposure of abundant adsorption sites and their electronic structural features thermodynamically favor more electron donation and promise superior reductive adsorption of PMs than other TMDs. Here, we report ultrahigh and selective PM extraction by using semimetallic TiS_2_ and TaS_2_ nanosheets, with TiS_2_ having *Q_e_* values of 8073, 2359 and 1154 mg/g for [AuCl_4_]^−^, [PdCl_4_]^2–^ and [PtCl_6_]^2–^, respectively, making them ideal candidates for the nearly complete recycling of PMs from electronic waste (e-waste), spent-fuel-cell catalysts and automotive catalytic converters (ACCs). In addition, our work highlights the efficient extraction of PM ions accompanied by the simultaneous formation of PM/TMD heterostructures, providing a synthetic pathway for the advanced catalysis and contact engineering of TMD electronic devices and opening up an avenue to address PM sustainability.

## RESULTS AND DISCUSSION

### Adsorption performance of semimetallic TiS_2_ and TaS_2_ nanosheets

The 1T-TiS_2_ and 2H-TaS_2_ nanosheets were exfoliated by the sonication of their thick flakes in a LiOH aqueous solution [41] and Raman analysis showed that the exfoliated nanosheets had no phase change during the process (Fig. S1 and Section S1) [42]. Transmission electron microscope (TEM) and atomic force microscope (AFM) analyses showed that both types of nanosheets had a lateral size of ∼2 μm and a thickness of 0.9–2.5 nm (Figs S2 and S3), suggesting a mono- and few-layer structure and a large specific surface area [43]. X-ray diffraction (XRD) and high-resolution TEM (HR-TEM) showed high crystallinity (Figs S2 and S4).

**Figure 1.**
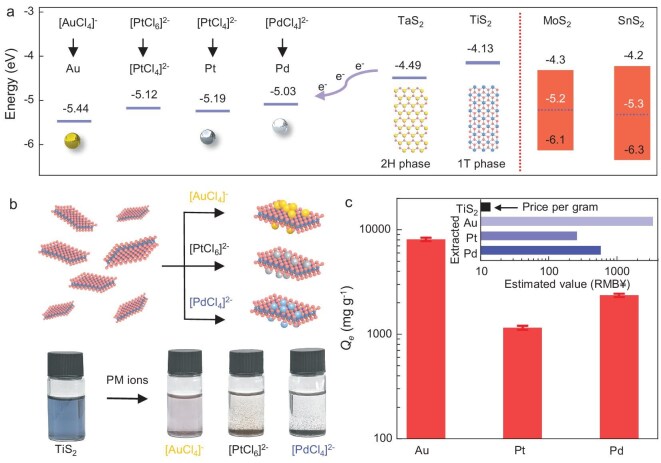
Highly efficient extraction of PMs. (a) Band alignment diagram for semiconducting SnS_2_ and MoS_2_, semimetallic TiS_2_, TaS_2_ nanosheets and [AuCl_4_]^−^/Au^0^, [PtCl_6_]^2–^/[PtCl_4_]^2–^, [PtCl_4_]^2–^/Pt^0^ and [PdCl_4_]^2–^/Pd^0^. Vertical bars span the bandgaps and the lines indicate the reduction potentials (for ions) [47, 48] and Fermi levels (for the TMD). (b) Schematic of the extraction process using semimetallic TMD nanosheets. After the TMD suspension was mixed with PM ions (100 ppm), the TMD suspension immediately changed its color from dark blue to clear. (c) Extraction capacity of TiS_2_ nanosheets for PM ions. Inset shows an economic analysis of the estimated PM recovery using TiS_2_. All data with error bars are presented as mean value ± standard deviation obtained from three samples.

The Fermi energy levels of TiS_2_ and TaS_2_ allowed their electron transfer to the [AuCl_4_]^−^, [PdCl_4_]^2–^ and [PtCl_6_]^2–^ ions (Fig. 1a), promising their strong adsorption for PM ions. An initial adsorption test showed that both TiS_2_ and TaS_2_ nanosheets had an immediate color change when mixed with [AuCl_4_]^−^, [PdCl_4_]^2–^ and [PtCl_6_]^2–^ solutions (initial concentration *C_0_* = 100 ppm), respectively, suggesting their strong interaction with and rapid adsorption of the PM ions (Fig. 1b). Taking 1T-TiS_2_ as an example, we measured the *Q_e_* values of [AuCl_4_]^−^, [PdCl_4_]^2–^ and [PtCl_6_]^2–^ (*C_0_* ∼100 ppm) as 8073, 2359 and 1154 mg/g, respectively (Fig. 1c). Given that TiS_2_ was the lightest and also possibly the cheapest TMD (∼14 RMB/g, estimated including the cost of pristine unexfoliated TiS_2_ bulk and its exfoliation incurred cost, details are described in Section S2) [38,44], its exceptionally high *Q_e_* of ∼8 g/g to high-value gold ions is particularly remarkable. Compared with the conventional adsorbents, including activated carbon (AC) and silica-based adsorbents (Section S2) [19,20,45,46], the measured ultrahigh *Q_e_* values of TiS_2_, with its intermediate cost, offer a high performance-to-cost ratio over these benchmark adsorbents, suggesting its strong economic viability for PM recycling (Fig. 1c, inset).

**Figure 2.**
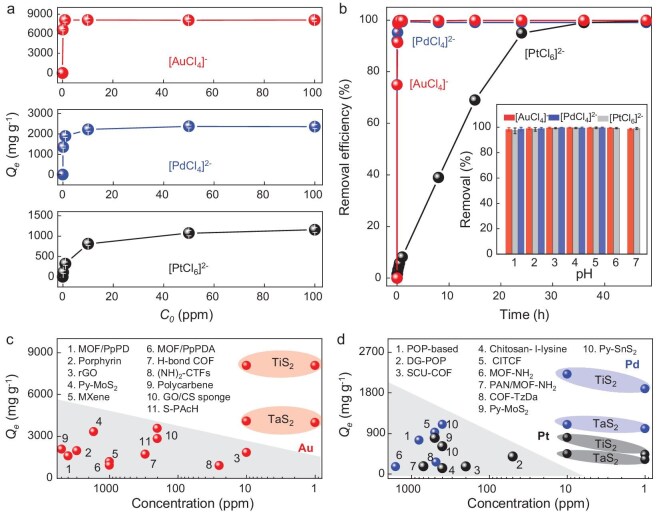
Adsorption performance of TiS_2_ and TaS_2_ nanosheets. (a) Extraction capacities of TiS_2_ for Au, Pt and Pd at a *C_0_* of 0.1–100 ppm. All data with error bars are presented as mean value ± standard deviation obtained from three samples. (b) Removal efficiency of TiS_2_ for Au, Pt and Pd at *C_0_* = 10 ppm. Inset shows the removal efficiency of TiS_2_ for Au, Pt and Pd in solutions with multiple pH values. Comparison of (c) Au, (d) Pt, and Pd extraction performance of semimetallic TMD nanosheets with other adsorbents. The data in the shaded area are taken from previously reported adsorbents listed in the figure and Table S2.

We next quantified the adsorption behavior of the TMD for PM ions. The *Q_e_* values for PM ions with *C_0_* varying from 0.1 to 100 ppm (Fig. 2a) were studied. Specifically, TiS_2_ had a *Q_e_* of 6620 mg/g to 0.1 ppm [AuCl_4_]^−^ and then became saturated at ∼8000 mg/g when the *C_0_* increased to 1 ppm. We also compared the *Q_e_* of TiS_2_ with and without light illumination and found that the light illumination had no detectable influence on the adsorption performance, aligning with its electronic structure, as depicted in Fig. 1a. Note that such a *Q_e_* at a low concentration of [AuCl_4_]^−^ was significantly higher than that of previously reported gold adsorbents, as summarized in Fig. 2c, promising its application for gold recycling at minute concentrations. For [PdCl_4_]^2–^ and [PtCl_6_]^2–^, the *Q_e_* started to saturate when the *C_0_* was ≥10 ppm; specifically, the *Q_e_* for [PdCl_4_]^2–^ was 1892, 2217 and 2359 mg/g and that for [PtCl_6_]^2–^ was ∼323, 813 and 1154 mg/g when *C_0_* = 1, 10 and 100 ppm, respectively. The adsorption kinetics (Fig. 2b) showed that TiS_2_ extracted >99% of the Au and Pd within 10 min (*C_0_* = 10 ppm)—significantly faster than the value for rGO that we reported previously [32]. In contrast, the adsorption of [PtCl_6_]^2–^ was slow, with only 3.2% of [PtCl_6_]^2^^–^ extracted in 10 min, and 68% and >95% removal efficiencies were achieved for 15 and 24 h of adsorption. We further fitted the adsorption kinetics with pseudo-first-order and pseudo-second-order kinetic models (Section S2, Table S1 and Fig. S5) [9] and found that the adsorption of [AuCl_4_]^−^ and [PdCl_4_]^2–^ fit with the pseudo-second-order model, with *R*^2^ values of 0.9996 and 0.9999, respectively, indicating a chemisorption mechanism, while [PtCl_6_]^2–^ fits a pseudo-first-order model, with *R*^2^ of 0.9889, suggesting adsorption-site-controlled kinetics, which will be discussed later in this study. The thermodynamics of the energy changes obtained on temperature-dependent adsorption performance suggest that all PM adsorptions and reductions are endothermic with a calculated positive enthalpy and indicate that the chemisorption of PMs can be further enhanced at a higher temperature (Figs S6 and S7).

We also investigated the influence of pH on *Q_e_* and removal efficiency. According to the Pourbaix diagram, which describes ion stability at different pH values, to make sure the measured *Q_e_* accurately reflected the adsorption of the PM ions to their original form, the pH ranges for stable [AuCl_4_]^−^ and [PtCl_6_]^2–^ were ∼1–7 and that for [PdCl_4_]^2–^ was <5 [30,49]. We therefore studied the *Q_e_* value in these pH ranges (Fig. S8). For [AuCl_4_]^−^ (*C_0_* = 10 ppm), TiS_2_ had the highest *Q_e_* (∼8076 mg/g) at pH = 3–5 and decreasing or increasing the pH resulted in a lower *Q_e_*, which probably was due to a trade-off between the surface charge of the TiS_2_ nanosheets and the stability of [AuCl_4_]^−^ at different pH values (Section S2). For [PdCl_4_]^2–^, the *Q_e_* increased with the pH, while, for [PtCl_6_]^2–^, it remained relatively stable in the tested pH range (Section S2) [10,32]. Furthermore, TiS_2_ showed a ∼100% removal efficiency for the three types of PM ions (Fig. 2b, inset). Not only did we find an ultrahigh *Q_e_* for TiS_2_, but we also found that the TaS_2_ nanosheets were highly efficient for the extraction of the three PM ions; their *Q_e_* values for [AuCl_4_]^−^, [PdCl_4_]^2–^ and [PtCl_6_]^2–^ were 4100, 1100 and 452 mg/g (Fig. 2d)—slightly lower than that of TiS_2_. Nevertheless, as summarized in Fig. 2c and d and Table S2, both TMDs had significantly higher *Q_e_* values than the previously reported PM adsorbents, e.g. MXene, graphene, metal–organic frameworks, covalent organic frameworks and pyridine-modified disulfide [24–27,32–34,50], especially in the sub-ppm to tens of ppm ranges relevant to the practical recycling of PM-containing waste streams.

Not only can semimetallic TMD nanosheets be used for PM extraction, but their macro-assemblies also enable a highly efficient, continuous gold-extraction process. To demonstrate this, we compressed a TiS_2_ aerosol (obtained by freeze-drying of its dispersion) using a syringe or by hand to obtain a flattened TiS_2_ aerosol [9] and it was used as a filter for the direct and rapid capture of [AuCl_4_]^−^ ions from its solution (Section S2 and Figs S9 and S10). We also conducted TiS_2_ membrane-based continuous filtration for gold extraction. The result showed that a ∼1-um-thick membrane (∼11.34 cm^2^) can recover 1.25 L of 10 ppm [AuCl_4_]^−^ solution while maintaining 95% extraction efficiency, suggesting its scalability for continuous gold extraction.

### Adsorption mechanism

**Figure 3.**
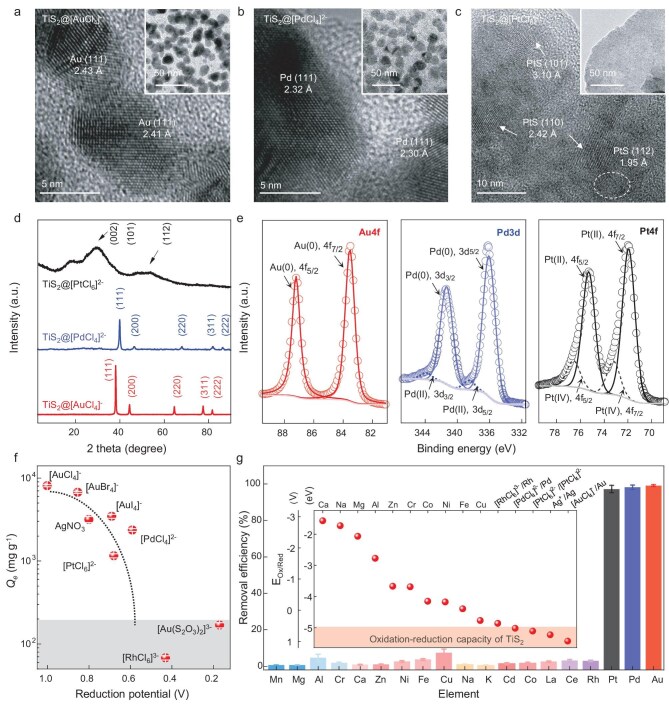
Structure analysis of adsorbed PM ions and TiS_2_ nanosheets, and the adsorption selectivity. TEM images of TiS_2_ nanosheets after adsorption of (a) [AuCl_4_]^−^, (b) [PdCl_4_]^2–^ and (c) [PtCl_6_]^2–^. (d) XRD patterns of TiS_2_ after adsorption of PM ions; the corresponding samples were denoted as TiS_2_@[AuCl_4_]^−^, TiS_2_@[PdCl_4_]^2–^ and TiS_2_@[PtCl_6_]^2–^. (e) XPS spectra of Au4f, Pd3d and Pt4f of TiS_2_@[AuCl_4_]^−^ (left panel), TiS_2_@[PdCl_4_]^2–^ (middle panel) and TiS_2_@[PtCl_6_]^2–^ (right panel). (f) Extraction capacity of TiS_2_ for PM ions with various reduction potentials. (g) Removal efficiencies of TiS_2_ for 15 types of interfering ions and 4 types of PM ions. Inset: redox potentials of the used metal ions. All data with error bars are presented as mean value ± standard deviation obtained from three samples.

To understand the efficient adsorption of PMs by semimetallic TMD, we used TEM (Figs. 3a–c and Figs S11 and S12), XRD (Fig. 3d) and X-ray photoelectron spectroscopy (XPS, Fig. 3e) to study TiS_2_@PM precipitates after adsorption. The TEM observation revealed the deposition of a high density of nanoparticles on the TiS_2_ nanosheets (particle size 3–18 nm). For [AuCl_4_]^−^ and [PdCl_4_]^2–^, the nanoparticles had lattice spacing consistent with the (111) plane of metallic Au and Pd [8,24,51], whereas the nanoparticles deposited after the adsorption of [PtCl_6_]^2–^ had lattice spacing corresponding to the (110) plane of PtS (Fig. 3c and Fig. S13) [52]. It was also worth noting that these nanoparticles were deposited on the adsorbent even for 10 min of adsorption (Figs S11 and S14), in good agreement with the observed rapid adsorption kinetics (Fig. 2b and Fig. S5). The XRD patterns (Fig. 3d) showed prominent peaks corresponding to elemental Au and Pd, and to PtS [52], consistently with the TEM observations. The deconvoluted XPS peaks of the PM deposited on TiS_2_ quantitatively showed that >99%, ∼92% and ∼82% of [AuCl_4_]^−^, [PdCl_4_]^2–^ and [PtCl_6_]^2–^ were reduced to Au(0), Pd(0) and Pt(II), respectively, with the rest of the adsorbed PM being Pd(II) and Pt(IV) (Fig. 3e and Fig. S15) [27,52,53]. Collectively, these results suggested that the dominant adsorption mechanism of PM by TiS_2_ was reductive adsorption and supported that the high Fermi level of semimetallic TMDs enables their efficient electron donation to PM ions [4,54]. We also noted that the reductive adsorption of [PtCl_6_]^2–^ yields PtS, which could be understood by the fact that the reduction of [PtCl_6_]^2–^ to Pt^0^ was a two-step process, with Pt^2+^ being an intermediate product, and its subsequent bonding to a sulfur site of TiS_2_ inhibited its further reduction to Pt^0^ because of the good chemical stability of PtS [55]. Nevertheless, because PtS was a primary ore for mining Pt, this suggested that PtS could easily be purified by using established methods [55].

Further insight regarding the reductive adsorption of PM ions by TiS_2_ was gained by measuring its *Q_e_* values for a series of PM ions with decreasing reduction potentials. As plotted in Fig. 3f, TiS_2_ has a *Q_e_* of >1100 mg/g for ions with reduction potentials of >0.59 V (versus a standard hydrogen electrode (SHE)), including [AuCl_4_]^−^, [AuBr_4_]^−^, AgNO_3_, [AuI_4_]^−^, [PdCl_4_]^2–^ and [PtCl_6_]^2–^, but a small *Q_e_* (<200 mg/g) for ions with reduction potentials of <0.59 V, such as [RhCl_6_]^3−^ and [Au(S_2_O_3_)_2_]^3−^, confirming the redox-reaction-driven reductive adsorption of PMs (Fig. S16). Such reduction potential-dependent extraction capacity also explains the differences in *Q_e_* for the three PM ions. The reduction potentials for [AuCl_4_]^−^/Au and [PdCl_4_]^2–^/Pd are 1.00 and 0.59 V, respectively. The smaller energy difference between [PdCl_4_]^2–^ and the Fermi level of TiS_2_/TaS_2_ makes electron donation from the TMD nanosheets less thermodynamically favorable for Pd compared with Au. Additionally, the smaller atomic weight of Pd (106 g/mol) versus Au (197 g/mol) also mathematically decreases the mass-based calculation of *Q_e_*. For [PtCl_6_]^2–^, its reduction potential ([PtCl_6_]^2–^/[PtCl_4_]^2–^) is ∼0.68 V, which is similar to that of Pd. Interestingly, as discussed previously, [PtCl_6_]^2–^ is reductively adsorbed as PtS—a semiconductor with a relatively low electrical conductivity. Therefore, the deposited PtS acts as a barrier, impeding electron transfer from TiS_2_ to subsequent [PtCl_6_]^2–^ ions, and hence a relatively low *Q_e_*. This is also in good agreement with adsorption kinetic analysis, as the formation of PtS reduces the available adsorption sites on the TiS_2_ surface, causing the adsorption kinetics to become diffusion-controlled, aligning with the observed pseudo-first-order kinetic model. It is worth noting that the high *Q_e_* observed for a wide range of gold ions expanded the potential use of TiS_2_ beyond the commonly seen [AuCl_4_]^−^ ion. For example, [AuI_4_]^−^, which is a product of the standard wet etching of a gold coating in the electronics industry, could be effectively recycled from wastewater by using TiS_2_, as evidenced by the high *Q_e_* of 3458 mg/g.

Not only is an ultrahigh *Q_e_* critical for adsorbents used in PM recycling, but also their PM-specific selectivity is of paramount importance. The redox-dominated adsorption also provided a way to selectively adsorb PM ions based on their reduction potentials. To validate this, we measured the PM-removal efficiency of TiS_2_ nanosheets in a simulated solution containing 15 interfering ions (∼100 ppm each of Na^+^, K^+^, Mg^2+^, Ca^2+^, Cd^2+^, Mn^2+^, Co^2+^, Cu^2+^, Zn^2+^, Ni^2+^, Al^3+^, Fe^3+^, Ce^3+^, La^3+^ and Cr^3+^) and four types of PMs (∼10 ppm each of Au^3+^, Pt^4+^, Pd^2+^ and Rh^3+^). The nanosheets showed a removal efficiency of >97% for Au^3+^, Pt^4+^ and Pd^2+^, but a low removal efficiency of <8% for the remaining ions, including Rh^3+^ (Fig. 3g), promising good PM selectivity for real-world PM recycling involving complex ion mixtures. We also quantified the *Q_e_* of TiS_2_ for Cu^2+^ and Ni^2+^ ions (100 ppm) and found that they were 260 and <10 mg/g, respectively, highlighting its good selectivity for PMs. In addition, the work function of TiS_2_ was –3.89 eV as measured by using ultraviolet photoelectron spectroscopy (Fig. S17), consistently with the reported value [38], whereas the reduction potentials (*E*_Red_ (vs SHE), Fig. S18) of [PdCl_4_]^2–^ and [RhCl_6_]^3−^ were +0.59 and +0.43 V, respectively, which translated into *E*_Red_ values of –5.03 and –4.87 eV (*E*_Red_ = –*E*_Red_ (vs SHE) – 4.44) [47,48], respectively. Note that TiS_2_ had a high *Q_e_* for [PdCl_4_]^2–^ but was incapable of adsorbing [RhCl_6_]^3−^, which indicated that the energy barrier required for the efficient adsorption by TiS_2_ should be ∼1–1.14 eV (a schematic is provided in Fig. S18 for comparison). This was in good agreement with previous results [4,34] and was explained by the energy needed for ion diffusion, ion desolvation and, in our case, electron donation and crystal nucleation and growth.

We next investigated why TiS_2_ had such a high *Q_e_* by using gold as an example. We first compared the *Q_e_* values achieved by using thick unexfoliated and exfoliated TiS_2_ flakes. For *C_0_* = 100 ppm, unexfoliated TiS_2_ had a *Q_e_* for gold of 476.8 mg/g (Fig. S19)—nearly 10 times lower than that of the exfoliated nanosheets. Similar results were also found for exfoliated and unexfoliated TaS_2_ (Fig. S19). Because our HR-TEM and Raman analyses showed that the TiS_2_ nanosheets had a highly crystalline structure, such a large difference in *Q_e_* could not be caused by defects, but was due to an increased specific surface area, which provided abundant adsorption sites for achieving a high *Q_e_* [24,32].

**Figure 4.**
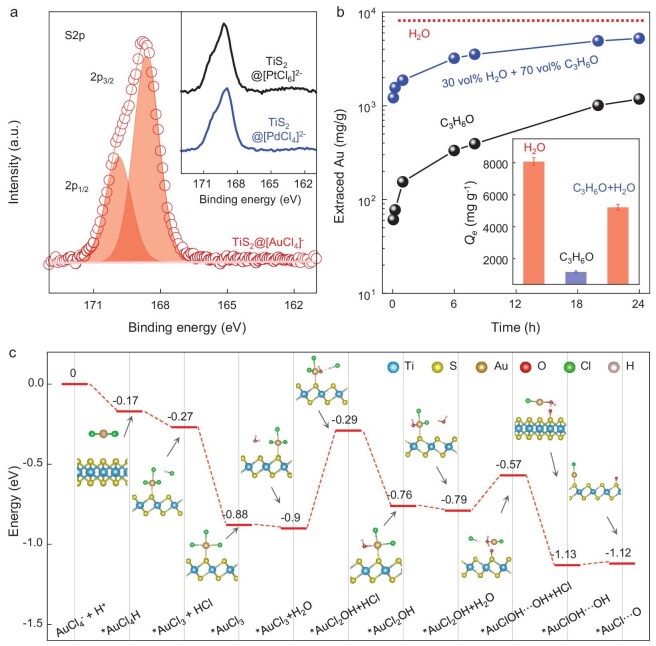
Understanding the role of H_2_O in the PM adsorption by TiS_2_. (a) Deconvoluted XPS S2p spectra of dissolved compounds collected after PM adsorption. The red line represents TiS_2_@[AuCl_4_]^−^. Inset shows the XPS S2p spectra of TiS_2_@[PdCl_4_]^2–^ and TiS_2_@[PtCl_6_]^2–^. (b) Extraction capacity as a function of time for [AuCl_4_]^−^ solutions using water, acetone and a 30 vol% water–70 vol% acetone mixture as the solvent. Inset summarizes *Q_e_* values for [AuCl_4_]^−^ measured using water, acetone and 30 vol% water–70 vol% acetone mixture as the solvent. All data with error bars are presented as mean value ± standard deviation obtained from three samples. (c) Calculated energy diagram of the early dechlorination reduction of TiS_2_ and Au species in an aqueous solution.

Furthermore, a *Q_e_* value of 8073 mg/g for [AuCl_4_]^−^ and the complete reductive adsorption of gold ions by TiS_2_ suggested that each TiS_2_ molecule donates >13 electrons during the adsorption process (calculation provided in Section S3); we therefore studied the active site that donates such a large number of electrons by analysing the structure change in the TiS_2_ nanosheets after adsorption. XPS analysis (Fig. S20) showed that the binding energies of Ti and S increased compared with pristine TiS_2_. The XPS spectra of Ti2p after adsorption were deconvoluted into two peaks (∼464.4 and 458.7 eV), which do not fit with the pristine Ti–S bond, but align with the typical Ti–O bond of TiO_2_ (Fig. S20) [56]. For sulfur, we measured the dissolved salt after adsorption and found that multiple binding energies at 168.6 and 169.8 eV were assigned to the sulfate species (Fig. 4a) [57,58]. This indicated that each TiS_2_ molecule could, in principle, donate no more than 16 electrons to the adsorbed [AuCl_4_]^−^, in quantitative agreement with the observed high *Q_e_*. XPS analysis (Fig. S21) on the structural changes in TiS_2_ during adsorption revealed sulfur oxidation (S^2–^ to S^6+^), with a decreasing S/Ti ratio from 2 to 0.63 for 10 min of adsorption, while no significant Ti loss was found, indicating the sulfur loss as sulfate releasing into the water (Section S3). The *Q_t_* calculated values, based on the lost sulfur donating the electrons needed for PM adsorption, were 3660, 5790 and 6300 mg/g at 1, 5 and 10 min, closely matching the experimental results (3540, 5470 and 6420 mg/g, Fig. S5), supporting that sulfur is the dominant electron-donation site for PM adsorption. Note that the XPS analysis also showed a similar transformation of the sulfur in TaS_2_ after [AuCl_4_]^−^ adsorption (Fig. S22), with the difference that a valency change in the tantalum (Ta^4+^→Ta^5+^) was also observed [59,60]. This suggested that one TaS_2_ molecule can donate no more than 17 electrons to [AuCl_4_]^−^; however, the observed slightly lower *Q_e_* for the adsorption of [AuCl_4_]^−^ by TaS_2_ must be attributed to the fact that its higher weight fraction than TiS_2_ reduces the weight-based PM-extraction capacity (the corresponding analysis is shown in Section S2).

It is important to note that, despite the oxidation of the sulfur of TiS_2_, an inductively coupled plasma (ICP) analysis indicated that <6 wt% of Ti is released into the solution (Table S3) and the structural tracking of individual TiS_2_ nanosheets during gold extraction suggests good structural integrity, maintained throughout the PM extraction without any structural collapse or dissolution (Fig. S14). This aligns with the retaining of structural integrity reported in a recent study on partially oxidized TMD nanosheets [61]. We attributed the structural stabilization to the surface encapsulation of the TMD nanosheets by deposited PM nanoparticles and incomplete electron donation (experimentally observed ∼13 electron donations per TiS_2_ molecule, compared with a theoretical value of 16), allowing PM ions to be selectively and reductively adsorbed onto the TiS_2_ surface. Collectively, these results provided unambiguous evidence that the sulfur of the TMD was the dominant site for extremely high electron donation, which was responsible for the ultrahigh *Q_e_* values.

The formation of sulfate species after adsorption suggested that oxygen was involved in the adsorption process, but was absent in the PM salt used; also, the good crystal structure of the TMD nanosheets (Fig. S2) did not explain the presence of such a large amount of oxygen after the reductive adsorption. It was therefore reasonable to suggest that the oxygen comes from the dissociation of the water solvent used in the adsorption. To testify to this, we first considered that the dissociation of water would lead to a release of protons in the solvent; indeed, the pH of the gold solution decreased from 3.96 to 3.77 after the gold extraction (Fig. S23). We then replaced the water with the nonprotonic solvent acetone and found that the *Q_e_* of the TiS_2_ nanosheets with an acetone solution of 100 ppm [AuCl_4_]^−^ was only ∼1180 mg/g (Fig. 4b and Fig. S24), which is ∼7 times lower than the *Q_e_* value measured in the water. In addition, adding 30 vol% of water to the acetone resulted in an increased *Q_e_* of 5217 mg/g. Because all the experiments were performed in the same ambient environment, the change in *Q_e_* produced by a change in solvent indicated that it was oxygen from the solvent rather than from the environment that dictated the PM adsorption. To further confirm that environmental oxygen did not contribute to the ultrahigh *Q_e_*, we performed gold extraction in an oxygen-free glovebox by using the same aqueous gold solution and found a similar *Q_e_* to the value obtained under ambient conditions, experimentally ruling out any role of atmospheric oxygen in the adsorption process.

To understand such an interesting role of water in the extraction process, we performed theoretical calculations by using spin-polarized density functional theory (DFT) and TiS_2_ crystal cells to evaluate the adsorption energy (*E*_ad_) of the [AuCl_4_]^−^ ion. Figure 4c shows the energy diagram of the dechlorination reduction process of [AuCl_4_]^−^ by TiS_2_ in an aqueous solution (Section S3). Firstly, the calculation indicated the favorable adsorption of [AuCl_4_]^−^ ions onto TiS_2_ (forming the ‘TiS_2_*AuCl_3_’ species) with an *E*_ad_ of –0.88 eV. This adsorption was accompanied by a dichlorination step, which made the gold atom directly bond to a sulfur atom of TiS_2_, forming an electron-donation site from sulfur to gold. Subsequently, a water molecule was adsorbed on the TiS_2_*AuCl_3_ intermediate, with the oxygen atom of water substituting one Cl^−^, followed by the dissociation of H_2_O, generating a TiS_2_*AuCl_2_OH intermediate in a thermodynamically favorable process with an *E*_ad_ of –0.76 eV, and forming a HCl byproduct, which explained the observed pH decrease after the gold extraction. Next, another water molecule approached the TiS_2_*AuCl_2_OH intermediate, initiating a second dechlorination reaction that resulted in the formation of the TiS_2_*AuClOH···OH intermediate, which involved the dissociation of the second water molecule due to the instability of two adjacent hydroxyl groups and then TiS_2_*AuClOH···OH transformed into TiS_2_*AuCl···O, accompanied by the formation of a S–O bond. The overall process led to the reduction of Au from Au(III) to Au(I), which had a higher reduction potential than Au(III) [48], and will convert to elemental Au by electron donation from the sulfur site and the formation of an S–O bond, in good agreement with the XPS results. The mechanism revealed by DFT showed that the S atom in TiS_2_ was the electron donor, while the entire process relied on the dissociation of the water molecule, which forms the S–O bond and releases HCl.

Taking these results together, we propose that water-mediated electron donation from the sulfur site of semimetallic TiS_2_ and TaS_2_ nanosheets is primarily responsible for the observed ultrahigh adsorption and deposition of PMs. The sulfur atoms of TiS_2_/TaS_2_, exhibiting an electron-rich character, are mostly converted into positive hexavalent sulfur (Fig. 4a), with such a strong electron-donation capability accompanied by the oxidation of sulfur, which is enabled by the water dissociation, providing oxygen to form sulfate species. In addition, the large surface area of the nanosheets allows efficient and rapid adsorption. Thermodynamically, the electronic structures of the TiS_2_ and TaS_2_ nanosheets (a narrow bandgap of 0.1–0.2 eV and a Fermi level that is at least ∼1 eV higher than the reduction potential of PM ions) allow electron donation from the nanosheets to the PM ions, making these semimetallic TMD nanosheets ideal for PM recovery.

### PM recovery from e-waste and catalyst scrap

**Figure 5.**
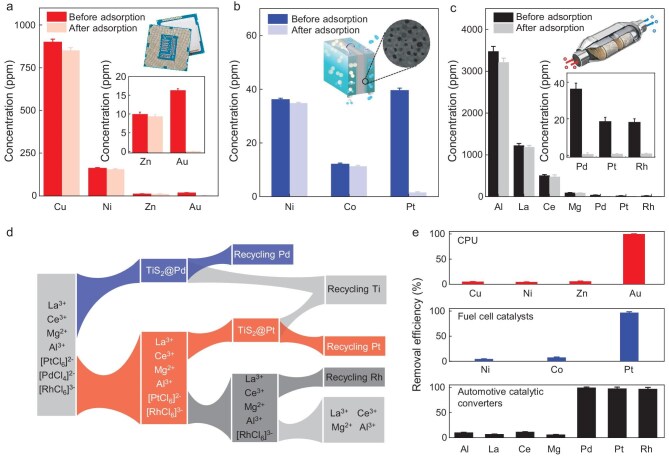
PM recovery from their corresponding wastes. Concentration changes of metal ions in (a) the CPU-board leaching solution, (b) the Pt/C catalyst leachate and (c) the ACCs leachate before and after recovery by the TiS_2_ nanosheets. (d) Schematic of the two-step PM-extraction process from the ACCs. (e) Corresponding removal efficiency of PM and coexisting metal ions by TiS_2_ nanosheets. All data with error bars are presented as mean value ± standard deviation obtained from three samples.

The observed ultrahigh *Q_e_* for PM ions, good selectivity and low cost of TiS_2_ (note that the cost of TaS_2_ is two orders of magnitude higher than that of TiS_2_) indicate that it can be used to recycle PMs from their corresponding waste. To demonstrate this, we recycled Au, Pd and Pt from their corresponding wastes, including e-waste, a Pd/C catalyst for chemical manufacturing, a Pt/C catalyst for fuel cells and scrap ACCs (experimental details in Section S4 and Fig. S25). For the e-waste (Fig. 5a and Fig. S25), TiS_2_ recovered >99% of the gold from the leachate of computer central processing unit (CPU) boards and showed negligible adsorption of the coexisting ions, including Cu^2+^ and Ni^2+^ (Fig. 5a). For the leachate of the Pd/C catalyst, TiS_2_ recovered 99% of the Pd (Section S4). For the Pt/C catalyst from scrap fuel cells (Fig. 5b), in which Co or Ni is frequently used as a co-catalyst, the TiS_2_ could directly recover 95.5% of the Pt from the leachate of the scrap catalyst while absorbing only 6.9% and 5.4% of Co^2+^ and Ni^2+^, respectively.

The spent ACCs (Fig. S25) had a complex composition containing Pd, Pt and Rh catalysts typically supported on ceramic substrates (e.g. MgO_2_, Al_2_O_3_, SiO_2_). In our case, after acid digestion of the ACCs, the amounts of Pd^2+^, Pt^4+^ and Rh^3+^ were 36.3, 18.5 and 18.1 ppm, respectively, while those of the coexisting Al^3+^, La^3+^, Ce^3+^ and Mg^2+^ ions were ∼3475, 1222, 501 and 89 ppm, respectively (Fig. 5c). Differently from the three types of PM-containing wastes mentioned previously, Pd and Pt co-exist in the ACCs and require selective adsorption to separate them. Because TiS_2_ had much faster adsorption kinetics of Pd than Pt (Fig. 2b), we believed that these two PMs could be separated based on their distinct adsorption kinetics. As shown in Fig. 5d, a two-step extraction process for their recovery and separation was designed. We added TiS_2_ nanosheets to the leachate to adsorb Pd^2+^ for 10 min and then removed them, resulting in the recovery of 99% of the Pd but only 8% of the Pt. We then added a second batch of TiS_2_ to extract the Pt for 36 h and 97% of the Pt was recovered with no detectable Pd observed in the TiS_2_. This efficient recovery of the Pd and Pt allowed further extraction of the Rh. We used iron powder to initiate a replacement reaction, which recovered 98% of the Rh, leaving the rest of the coexisting ions in the leachate (Fig. 5e). These steps allowed the selective recovery of ∼90% to ∼100% of the Pd, Pt and Rh.

Following the previous report [62], the TiS_2_ with the adsorbed PMs (including those recycled from scrap CPUs, Pd/C catalysts, Pt/C catalysts and ACCs) was dissolved in aqua regia and chemically reduced to separate the PM from the insoluble oxidized Ti-containing precipitates through a syringe filter with a 0.22-µm pore-size polyethersulfone (PES) membrane. Energy dispersive spectroscopy (EDS) results showed that the purities of the recycled Au, Pt and Pd from their corresponding scrap CPUs, Pt/C catalysts and Pd/C catalysts all were >97 wt% (Fig. S26)—significantly higher than those achieved by AC and ion exchange resins (using Au recovery as an example) [63]. For the recycled Pd and Pt from ACCs, the Pd purity of the product after the first purification step was ∼88 wt%, with ∼11 wt% of Pt, and the Pt purity of the product after the second step was ∼98 wt%, respectively (Fig. S26).

EDS elemental analysis showed that the precipitates after the separation of PMs primarily consisted of titanium (61 wt%) and oxygen (33 wt%), with the remaining being carbon (Fig. S26). These precipitates were then regenerated into TiS_2_ (Fig. S27) by using a flash heating (∼973 K, 10 s, six cycles) process under a CS_2_ flow. The regenerated TiS_2_ exhibited a retained extraction capacity of 6700 mg/g, demonstrating true reusability for PM recovery. Notably, this regeneration required energy of as low as 38.4 kJ, which is ∼0.22% of that of conventional electric furnace regeneration and 0.92% of the total energy for TiS_2_ exfoliation and PM adsorption energy (Section S5).

Finally, by using AC as a benchmark counterpart [64], analysis of the environmental impact and carbon emissions of the proposed TiS_2_-based gold-recovery method (Section S5) demonstrated that it not only offers strong economic viability, but also has significantly lower environmental and carbon impacts, which can be attributed to the ultrahigh adsorption capacity of TiS_2_ and its environmentally benign exfoliation process, strongly supporting its feasibility for future engineering applications.

## CONCLUSIONS

We have discovered that semimetallic TiS_2_ and TaS_2_ nanosheets are highly efficient for the adsorption of PM ions, including Au, Pd and Pt. Benefitting from a near-zero bandgap and an appropriate Fermi energy level, these nanosheets donate ∼13 electrons/molecule to the PM ions primarily from their sulfur sites, resulting in an ultrahigh *Q_e_* and excellent adsorption selectivity. We have also demonstrated the use of TiS_2_ nanosheets for the recovery of PMs from their wastes, including e-waste, scrap catalysts and ACCs, promising their use for PM recycling. Our study has revealed complicated electron-donation behavior from the adsorbent to the ions that is a result of the interplay between the adsorbate, adsorbent and solvent, which provides insight for designing novel adsorbents. Furthermore, the observed aqueous-phase PM deposition on the TiS_2_ and TaS_2_ nanosheets provides a new strategy and insight for the surface functionalization of TMD nanosheets, which are interesting for the interfacial engineering of TMD-based electronic devices. In addition, as PM nanoparticles have broad applications in various fields, particularly in catalysis, we envisage that recycled PM nanoparticles that can be further finely tuned by adjusting the adsorption time and PM ionic concentration hold great potential for the reuse and upcycling of recycled PMs as high-value catalysts. Given the irreplaceable role of PMs in modern industry, our finding opens up a way to use 2D materials to address global PM sustainability.

## METHODS

### Extraction capacity of TiS_2_ and TaS_2_ nanosheets for PM ions

A KAuCl_4_ aqueous solution was mixed with a TiS_2_ or TaS_2_ suspension (the concentration varied from 0.1 to 10 mg/mL and a had insignificant influence on the resulting *Q_e_*) to form mixtures with initial gold concentrations (note that the elemental concentrations used in this study were similar to those in the previous reports) of 0.1, 1, 10, 50 and 100 ppm [32]. The pH values of the solutions were adjusted with 0.1 M HCl or NaOH solutions. The weight ratio between the Au ions and the TiS_2_/TaS_2_ was 10:1, optimized according to the *Q_e_*, which allows excessive Au ions for extraction, and these mixtures with a fixed volume of ∼100 mL were stirred for 24 h at 25°C to determine the extraction capacity of the nanosheets. After extraction, the mixtures were filtered through a syringe filter with a 0.22-µm pore-size PES membrane and then the ion concentrations were determined by using inductively coupled plasma optical emission spectroscopy (ICP-OES). Finally, the extraction capacity (*Q_e_*, mg/g) was calculated by using the following equation: *Q_e_ =* (*C_0_* *–* *C_e_*) *·V/m*, where *C_0_* (mg/L) and *C_e_* (mg/L) represent the initial concentration and equilibrium concentrations, respectively, and *V* (L) and *m* (g) represent the volume of the solution and the mass of the TMD nanosheets, respectively.

To determine the *Q_e_* of TiS_2_ for the [AuBr_4_]^−^, [AuI_4_]^−^ (obtained by following the previous report to mix [AuCl_4_]^−^ with KI solution [65]), [Au(S_2_O_3_)_2_]^3−^, [RhCl_6_]^3−^ and Ag^+^ (from AgNO_3_) ions, we used the same procedure as that for [AuCl_4_]^−^, with an initial ion concentration of 100 ppm. During the extraction, the pH was ∼3–4, except for [Au(S_2_O_3_)_2_]^3−^, which was conducted at a pH of ∼10, due to its known instability in acidic solutions [32]. A similar procedure was used to obtain the extraction capacity for Pd and Pt from their corresponding ions ([PdCl_4_]^2–^ and [PtCl_6_]^2–^) and the weight ratios of the Pd and Pt ions to the TiS_2_ or TaS_2_ were set at 3:1 and 2:1, respectively.

In our single metal-extraction tests, we used an aqueous solution of CuCl_2_, NiCl_2_ to achieve 100-ppm concentrations of Cu^2+^, Ni^2+^. The weight ratio of each metal ion and the TiS_2_ was 1:1.

### Selective extraction for PMs from a multicomponent solution

To determine the extraction selectivity of TiS_2_, TiS_2_ nanosheets (5 mg) were added to the 100 mL of solution (pH = ∼2) containing Au^3+^ (∼10 ppm), Pt^4+^ (∼10 ppm), Pd^2+^ (∼10 ppm) and Rh^3+^ (∼10 ppm), as well as 15 interfering metals (∼100 ppm for each ion), including Na^+^, K^+^, Mg^2+^, Ca^2+^, Cd^2+^, Mn^2+^, Co^2+^, Cu^2+^, Zn^2+^, Ni^2+^, Al^3+^, Fe^3+^, Ce^3+^, La^3+^ and Cr^3+^. After being stirred for 24 h at 25°C, the mixture was filtered and the ion concentrations in the filtrate were analysed by using ICP-OES and used to calculate the removal efficiency (Section S2).

### Characterization

Powder XRD patterns were obtained by using a Rigaku MiniFlex600 X-ray diffractometer with Cu Kα radiation. High-resolution TEM images of TiS_2_, the TaS_2_ nanosheets and TiS_2_@[AuCl_4_]^−^, TiS_2_@[PdCl_4_]^2–^ and TiS_2_@[PtCl_6_]^2–^ were obtained by using a double spherical aberration-corrected microscope (Spectra 300) or an FEI Tecnai G2 F30 transmission electron microscope. The thickness of the TMD nanosheets was measured by using AFM (Bruker Dimension Icon). Raman spectroscopy measurements of the TMD nanosheets was performed under ambient conditions via a Thermo Fisher DXR2xi Raman imaging microscope using a 532-nm laser. XPS spectra were obtained by using a Thermo Fisher ESCALAB Xi+ equipped with Al Kα radiation. The work functions of the TMD nanosheets were measured by using UPS (Thermo Fisher Nexsa). The concentration of the metal ions was analysed by using ICP-OES (SPECTRO ARCOS Ⅱ MV) and a Ultraviolet–visible spectrophotometer (JASCO V760).

## Supplementary Material

nwaf522_Supplemental_Files
