# Supplementary material for: Water-mediated recycling of gold, palladium and platinum using semimetallic TiS2 and TaS2 nanosheets
Source: Natl Sci Rev. 2025 Nov 20;13(1):nwaf522. doi: 10.1093/nsr/nwaf522 (PMC12796814; doi:10.1093/nsr/nwaf522)
Supplement: nwaf522_Supplemental_Files [file nwaf522_supplemental_files.zip › Supplementary data.pdf]

# Supplementary Information

## Water-mediated recycling of gold, palladium, and platinum using semimetallic $TiS_2$ and $TaS_2$ nanosheets

Jianhong Wei[1,#], Miaofei Huang[1,#], Kuang Yu[1,*], Huanjing Liang[1], Fei Li[1], Kaiqiang Zheng[1], Fangluo Chen[1], Yibo Gao[1], Yang Su[1,*] and Hui-Ming Cheng[2,3,4,*]

[1]Institute of Materials Research, Shenzhen International Graduate School, Tsinghua University, Shenzhen 518055, China;

[2]Institute of Technology for Carbon Neutrality, Shenzhen Institutes of Advanced Technology, Chinese Academy of Sciences, Shenzhen 518055, China;

[3]Faculty of Materials Science and Energy Engineering, Shenzhen University of Advanced Technology, Shenzhen 518055, China;

[4]Shenyang National Laboratory for Materials Science, Institute of Metal Research, Chinese Academy of Sciences, Shenyang 110016, China

***Corresponding authors**. E-mails:

yu.kuang@sz.tsinghua.edu.cn; su.yang@sz.tsinghua.edu.cn; hm.cheng@siat.ac.cn

[#]Equally contributed equally to this work.

**Supplementary Section 1 | Exfoliation of $TiS_2$ and $TaS_2$ nanosheets and their structure analysis**

Transition metal dichalcogenides (TMDs), including $TiS_2$ and $TaS_2$ nanosheets, were exfoliated following a previous study[1]. Briefly, the 1T-$TiS_2$ bulk flakes (0.2 g, Nanjing NXNANO Tech. Co., Ltd.) were mixed with 4 mL of 2 M LiOH aqueous solution and stirred for 1 hour under nitrogen. The intercalated 1T-$TiS_2$ powder was washed several times with deionized water until the pH was ~7. It was then sonicated for 30 min in an ice bath to obtain a dispersion of 1T-$TiS_2$ nanosheets. The dispersion was centrifuged at 3000 rpm for 10 min, and the supernatant was collected and used for the extraction test. We note that the $TiS_2$ aqueous dispersion was stored at 5°C, and no obvious difference in extraction capacity was observed between freshly exfoliated $TiS_2$ nanosheets and those stored for 7 days. Furthermore, freeze-drying and vacuum-packaging the nanosheets after exfoliation enable extended storage, with no obvious difference in extraction capacity observed between freshly prepared $TiS_2$ nanosheets and those stored in dry form for 30 days. We used an identical procedure for the exfoliation of the 2H-$TaS_2$ nanosheets.

After exfoliation, the TMD nanosheets were analyzed using zeta potentials, Raman, transmission electron microscope (TEM), atomic force microscope (AFM), and X-ray diffraction (XRD). The zeta potentials of $TiS_2$ and $TaS_2$ suspensions exceeded -40 mV, indicating good colloidal stability, while the agglomeration happens once the TMD and PM solution were mixed, as the metal ions will neutralize the surface charge of TMD, similar to most of colloid-based adsorption, a record high extraction capacity at low concentration suggests agglomeration was likely soft agglomerate which does not influence the accessible surface area for efficient precious metal (PM) adsorption. For the Raman analysis (Fig. S1), similar to its bulk materials, the characteristic Raman peaks corresponding to $A_{1g}$ (333 $cm^{-1}$) and $E_g$ (227.2 $cm^{-1}$) vibration modes of 1T-$TiS_2$[2,3], and peaks at the 204 $cm^{-1}$, 279 $cm^{-1}$, and 394 $cm^{-1}$ due to the $E_{1g}$, $E^1_{2g}$, and $A_{1g}$ lattice vibration modes of 2H-$TaS_2$ were evident[4]. There was no peak for other crystalline phases, suggesting the predominant phases of $TiS_2$ and $TaS_2$ were 1T and 2H, respectively.

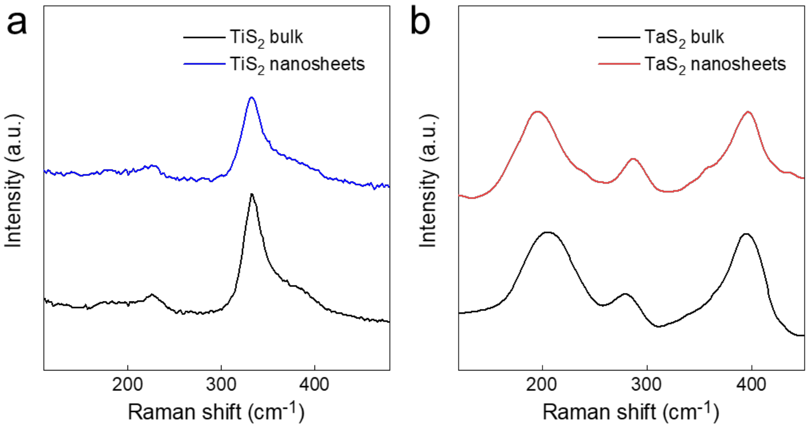


**Fig. S1** Raman spectra of bulk and exfoliated (a)$TiS_2$ and (b)$TaS_2$ nanosheets

Fig. S2 showed TEM images of representative exfoliated 1T-$TiS_2$ and 2H-$TaS_2$ nanosheets were 2D features with wrinkles, indicating successful exfoliation. The high-resolution TEM images clearly showed that the $TiS_2$ nanosheet had a lattice spacing of 0.267 nm, corresponding to its (101) crystal plane[5, 6], and the $TaS_2$ nanosheet had a lattice spacing of 0.290 nm, corresponding to its (100) crystal plane, the high-resolution TEM images showed high crystalline structure[7, 8], and the selected area electron diffraction (SAED) patterns of 1T-$TiS_2$ and 2H-$TaS_2$ showed no impurities, suggesting the good crystalline structure of the nanosheets.

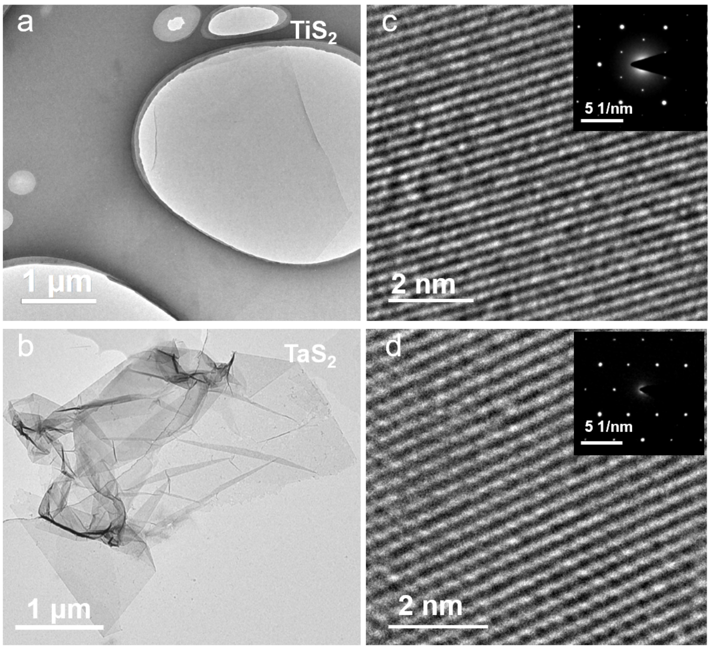


**Fig. S2** TEM analysis of exfoliated TMD nanosheets. Low-magnitude images of (a) $TiS_2$ and (b) $TaS_2$ nanosheet. High-resolution TEM images of (c) $TiS_2$ and (d) $TaS_2$ nanosheets, and insets were their corresponding SAED patterns.

AFM analysis (Fig. S3) showed that the exfoliated nanosheets had a lateral size of 1-3 μm, and the thicknesses of both were in the range of 0.9 - 2.5 nm, and in good agreement, the TEM images of the edges of the TMD nanosheets further support that the exfoliated nanosheets were mono- or few-layer.

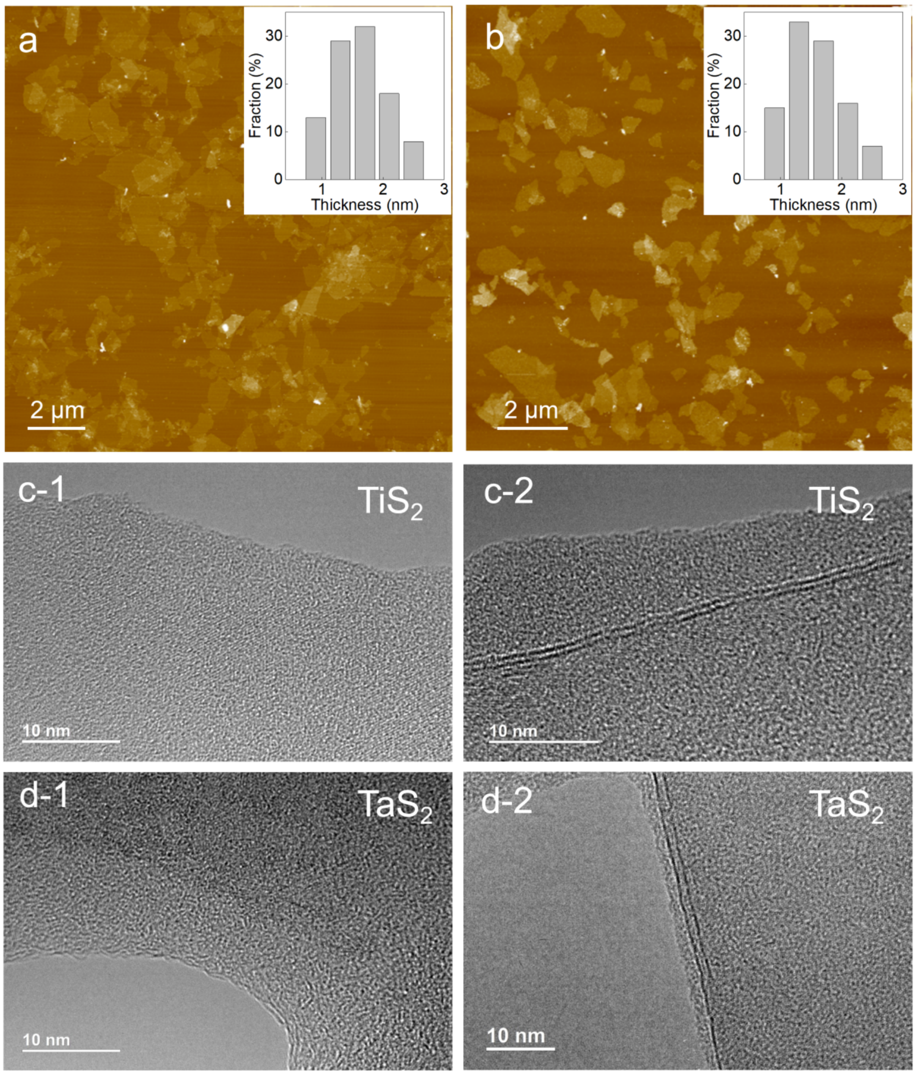


**Fig. S3** Thickness of the exfoliated nanosheets. (a-b) AFM image of exfoliated (a) 1T-$TiS_2$ and (b) 2H-$TaS_2$ nanosheets. (c-d) TEM images of exfoliated (c) 1T-$TiS_2$ and (d) 2H-$TaS_2$ nanosheets, with their edges showing mono- and few-layered structures.

For the XRD analysis, the patterns (Fig. S4) showed that the peaks of laminates assembled from the exfoliated $TiS_2$ (001) and $TaS_2$ (002) were slightly shifted to a lower angle compared to their corresponding powders, suggesting lattice expansion and successful exfoliation. The XRD patterns in Fig. S4 showed diffraction peaks of $TiS_2$ and $TaS_2$ at low angles located <10 degrees, these peaks could be assigned to water-intercalated $TiS_2$ and $TaS_2$ laminates, similar to the previously reported[1,7-8].

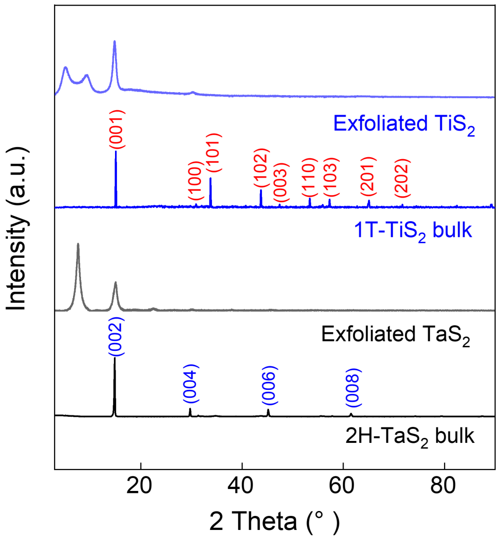


**Fig. S4** XRD patterns of laminates assembled from exfoliated ($TiS_2$ and $TaS_2$) nanosheets and their bulk powders.

## Supplementary Section 2 | PM extraction by $TiS_2$ and $TaS_2$ nanosheets

2.1 Economic analysis for PM extraction by $TiS_2$

The economic estimated value of recycled PM by per gram $TiS_2$ was calculated using Eq. (1).

$$Estimated\ \ value = Q_e \times PR \qquad \text{Eq. (1)}$$

where $Q_e$ was the extraction capacity (g/g), and PR was the market price (RMB/g) of precious metals. The market prices for Au, Pd, and Pt in 2024 were approximately 420, 245, and 224 RMB/g.

For the cost of $TiS_2$ nanosheet, our calculation was based on the following breakdown: Preparing 1 g of exfoliated $TiS_2$ requires ~1 g of bulk $TiS_2$ particulate (13 RMB/g), ~1 g of LiOH (~0.1 RMB/g), 20 mL of water, and energy for magnetic stirring. Given that the cost of bulk $TiS_2$ dominates, we estimate an additional 1 RMB/g for exfoliation (covering LiOH, water, and energy), resulting in a total cost of ~14 RMB/g.

Regarding cost-efficiency comparison, we agree that comparing material costs and efficiency against benchmark adsorbents (e.g., activated carbon, silica-based materials, functionalized/d polymers) will strengthen industrial applicability discussions, and we will incorporate such analysis. Literature reports ~50% efficiency after 2 hours and <100 mg/g capacity[9, 10], with a cost of ~0.014 RMB/g for industrial-scale activated carbon, silica-based adsorbents report 40-275 mg/g[11], with a cost of >70 RMB/g for

silica-based adsorbent (SBA-15, msesupplies.com). In contrast, our $TiS_2$ achieves >99% efficiency in 10 minutes with a capacity of ~8000 mg/g. At 14 RMB/g, $TiS_2$ offers a superior performance-to-cost ratio over these benchmarks.

2.2 kinetics and thermodynamics of adsorption

To study the possible effect of light on PM adsorption, we compared the extraction capacity of $TiS_2$ for 10 ppm $[AuCl_4]^-$ ions under natural environmental light and in the dark. The measured $Q_e$ values were both around 8000 mg/g, indicating that illumination has a negligible influence on the adsorption behavior. This is not surprising as semimetallic $TiS_2$ has a band gap of ~0.2 eV (Fig.1a), such little bandgap should not allow a strong light responsivity for reductive adsorption due to electron/hole recombination.

To evaluate the extraction kinetics of $TiS_2$ for $[AuCl_4]^-$, $[PdCl_4]^{2-}$, and $[PtCl_6]^{2-}$, we monitored the concentrations of precious metal ions with an initial concentration of 10 ppm in the solution at different time intervals using inductively coupled plasma optical emission spectrometry (ICP-OES). The mass ratios of PM to $TiS_2$ were set at 10:1 for Au, 2:1 for Pt, and 3:1 for Pd (see Methods), ensuring excess PM ions for accurate measurement of extraction capacity. The time-dependent extraction capacities were measured and analyzed with respective to time using two kinetic models: the pseudo-first-order model and the pseudo-second-order model (Fig. S5).

For the Pseudo-First-Order Model, the equation is expressed as (Eq. (2)):

$$\ln(Q_e - Q_t) = \ln Q_e - k_1 \cdot t \qquad \text{Eq. (2)}$$

Where, $Q_e$ and $Q_t$ are the extraction capacity at equilibrium and at time, respectively. $k_1$ is a pseudo-first-order rate constant. $t$ is extraction time. This model assumes that the kinetics of adsorption is influenced by the number of non-adsorbed sites present on the adsorbent's surface.

For the Pseudo-Second-Order Model (Eq. (3)), based on chemisorption mechanisms and governed by mass balance equations and second-order rate derivatives, the equation is:

$$\frac{t}{Q_t} = \frac{1}{k_2 \cdot Q_e^2} + \frac{t}{Q_e} \qquad \text{Eq. (3)}$$

where: $k_2$ is the pseudo-second-order rate constant.

As shown in Fig. S5 and Table S1, for the extraction kinetics of Au and Pd ions, the pseudo-second-order model exhibited higher fitting accuracy ($R^2$ values in Table S1), indicating that the extraction process aligns with chemisorption mechanisms. For extracting Pt ions, the pseudo-first-order model showed a better fit ($R^2$ values in Table S1), suggesting adsorption-site-controlled kinetics.

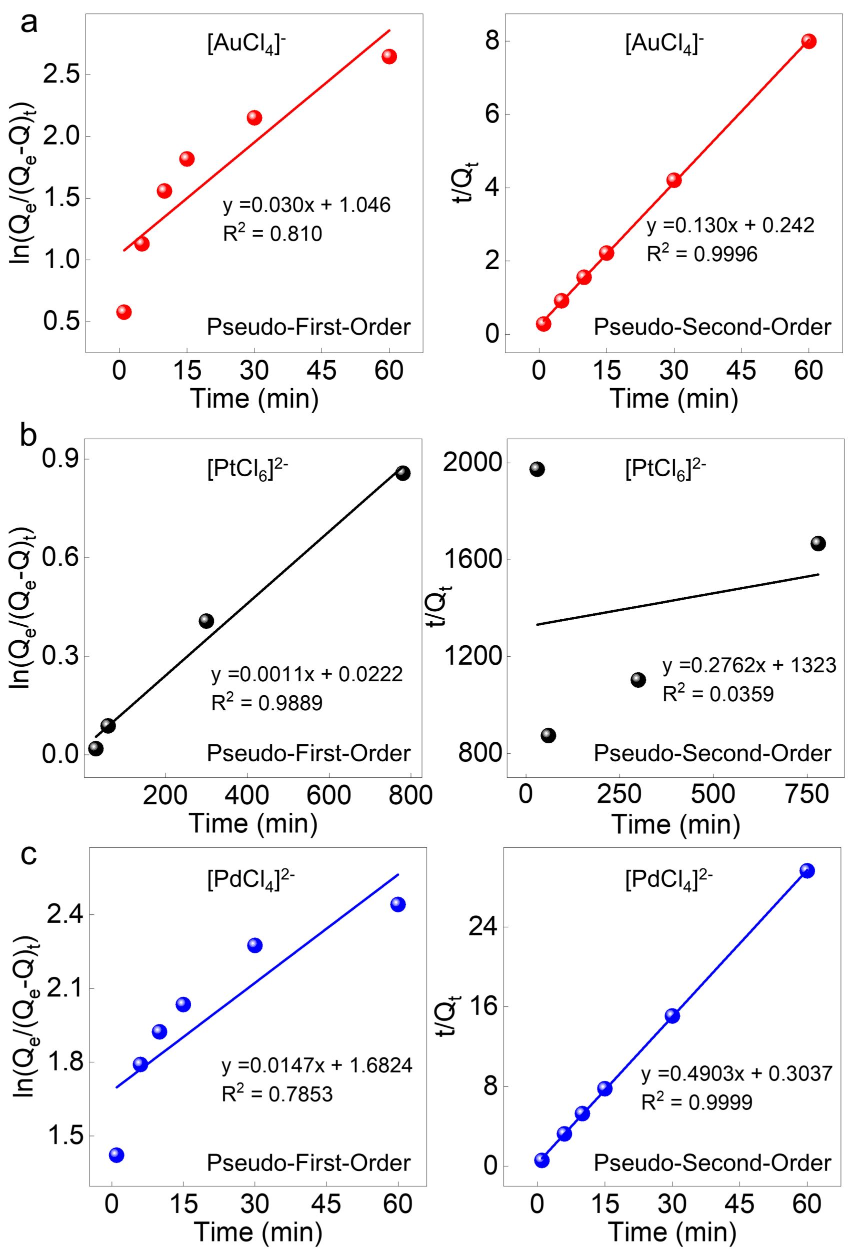


**Fig. S5** Extraction kinetics of (a) $[AuCl_4]^-$, (b) $[PtCl_6]^{2-}$, and (c)$[PdCl_4]^{2-}$ fitted with pseudo-first (left panel) and pseudo-second-order (right panel) kinetic models.

**Table S1.** Adsorption kinetic parameters from the fitted data.

| Exacting PM | Kinetic model | Parameter | $TiS_2$ nanosheets |
|---|---|---|---|
| $[AuCl_4]^-$ | Pseudo-first | $k_1$, $min^{-1}$ | 0.030 |
| | | $R^2$ | 0.810 |
| | Pseudo-second | $k_2$, g/g·min | 0.063 |
| | | $R^2$ | 0.9996 |
| $[PtCl_6]^{2-}$ | Pseudo-first | $k_1$, $min^{-1}$ | 0.0011 |
| | | $R^2$ | 0.9889 |
| | Pseudo-second | $k_2$, g/g·min | 0.00114 |
| | | $R^2$ | 0.0359 |
| $[PdCl_4]^{2-}$ | Pseudo-first | $k_1$, $min^{-1}$ | 0.0147 |
| | | $R^2$ | 0.7853 |
| | Pseudo-second | $k_2$, g/g·min | 0.67 |
| | | $R^2$ | 0.9999 |

We also studied the temperature-dependent adsorption behavior. We have measured the extraction capacities of PM (initial concentration of 10 ppm) by $TiS_2$ at elevated temperatures (5, 25, 45, and 60°C), as shown in Fig. S6, which showed increased extraction capacity with temperature, suggesting an endothermic adsorption. The increased extraction capacity with temperature was possible due to a temperature-enhanced diffusion rate for the PM ions, and also an increase in reductive reaction kinetics.

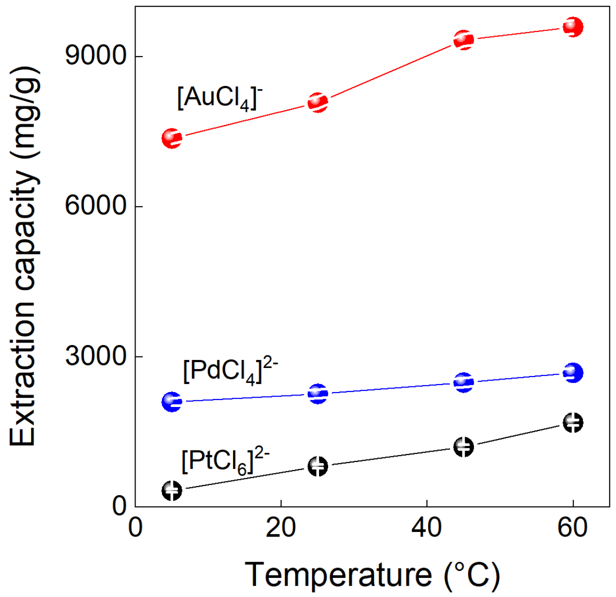


**Fig. S6** Temperature-dependent extraction capacity for PM by $TiS_2$ nanosheets.

To quantify the adsorption thermodynamics, the adsorption enthalpy ($\Delta H$) and Gibbs free energy $\Delta G$ were obtained from the following mathematical equation[12, 13]:

$$K_d = \frac{Q_e}{C_e} \qquad \text{Eq. (4)}$$

$$\Delta G = -RT \ln K_d \qquad \text{Eq. (5)}$$

$$\ln K_d = \frac{\Delta S}{R} - \frac{\Delta H}{RT} \qquad \text{Eq. (6)}$$

Where $K_d$ is the equilibrium adsorption distribution constant; $\Delta G$ (kJ/mol) is the Gibbs free energy change of adsorption; $\Delta H$ (kJ/mol) is the enthalpy change; $\Delta S$ (J/mol·K) is the entropy change; $C_e$ (mg/L) is the equilibrium solution concentration corresponding to extraction capacity $Q_e$ at temperature $T$ (K); and $R$ is the universal gas constant (8.314 J/(K·mol)).

$\Delta G$ was negative ($\Delta G < 0$) at all temperatures, indicating that adsorption was spontaneous. Plotting ln$K_d$ versus 1/T gave a straight line with slope (Fig. S7), which was equal to $\Delta H/R$. We then obtained $\Delta H$ values of 30.11, 43.73, and 17.89 kJ/mol for the adsorption of $[AuCl_4]^-$, $[PtCl_6]^{2-}$, and $[PdCl_4]^{2-}$ on $TiS_2$, respectively. $\Delta H$>0 indicated that the adsorption process was endothermic (i.e., absorbed heat during adsorption). This was consistent with the trend of extraction capacity increasing with temperature.

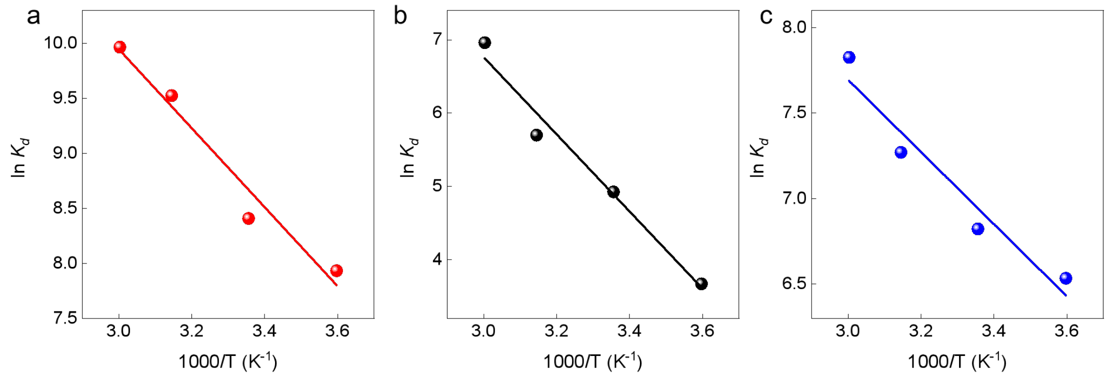


**Fig. S7** Thermodynamic parameters for the adsorption of (a) $[AuCl_4]^-$, (b) $[PtCl_6]^{2-}$, and (c)$[PdCl_4]^{2-}$ obtained from the van't Hoff equation.

2.3 pH-dependent extraction capacities for different PM ions by $TiS_2$ nanosheets

The suitable pH values for PM extraction were determined from the Pourbaix diagram following a previous report and were illustrated in the main text[14, 15]. The extraction capacity ($Q_e$) for Au, Pt, and Pd ions at different pH values was measured and plotted versus the corresponding pH values (Fig. S8). It was worth noting that, despite $Q_e$ changes with pH, it remained high, which was critical for a high and stable PM extraction.

For the extraction of $[AuCl_4]^-$, the $Q_e$ remained stable the pH was in the range of 3-5. The decrease at pH<3 could be attributed to the protonation of TMD nanosheets, eliminating certain adsorption sites, hence giving a lower $Q_e$[16-18], while, the $Q_e$ decrease at pH >5 was attributed to the hydrolysis of $[AuCl_4]^-$, forming a mixture of $[AuCl_4]^-$, $[AuCl_3(OH)]^-$, $[AuCl_2(OH)_2]^-$, $[AuCl_3(OH)_1]^-$ and $[Au(OH)_4]^-$[16, 20], leading to a decrease in the reduction potenial[20, 21], hence a lower $Q_e$ was observed. A similar phenomenon had been previously reported in the literature[16, 18, 21].

For the extraction of $[PtCl_6]^{2-}$, the $Q_e$ remained stable across the tested pH range 3-7, due to the stability of the Pt (IV) species within this range[14, 15]. The slight decrease in $Q_e$ observed for both $[PtCl_6]^{2-}$ and $[PdCl_4]^{2-}$ at low pH could be attributed to the protonation of the nanosheets, similar to the case of $[AuCl_4]^-$.

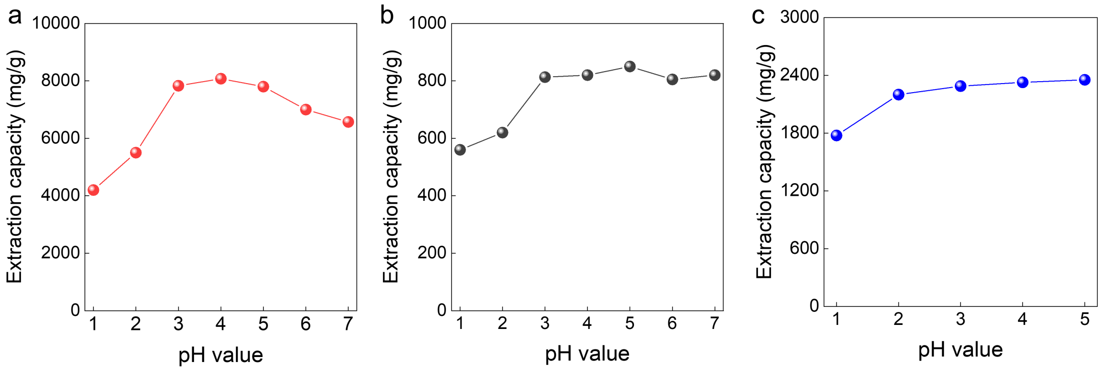


**Fig. S8** Extraction capacity of $TiS_2$ nanosheets at different pH values. $Q_e$ values of $TiS_2$ nanosheets for (a) $[AuCl_4]^-$, (b) $[PtCl_6]^{2-}$, and (c) $[PdCl_4]^{2-}$ ions at 10 ppm.

2.4 Removal efficiencies of different PM ions with different pH by $TiS_2$ nanosheets

To determine the PM removal efficiency of $TiS_2$ nanosheets, we used an initial PM concentration of 10 ppm, and the weight ratios between the Au, Pd, and Pt ions to $TiS_2$ were set at 3:1, 1:1, and 0.1:1, respectively. The mixture at different extraction time was sampled to study the removal kinetics. The removal efficiency (R) was calculated using Eq. (7).

$$R = \frac{(C_0 - C_e)}{C_0} \times 100\% \qquad \text{Eq. (7)}$$

where $C_0$ (mg/L) and $C_e$ (mg/L) were the initial concentration and equilibrium concentrations, respectively, and were determined using inductively coupled plasma optical emission spectroscopy (ICP-OES).

The removal efficiencies at different pH values were also determined using a similar procedure by changing the extraction pH using NaOH or HCl.

2.5 Comparison of $Q_e$ achieved by $TiS_2$ and $TaS_2$ nanosheets with previous reports.

**Table S2:** Extraction capacities of various gold adsorbents.

| Metal ion | Adsorbent | $C_0$ (ppm) | $Q_e$ (mg/g) | Ref. |
|---|---|---|---|---|
| **Au(III)** | rGO | 10 | 1850 | 22 |
| **Au(III)** | MOF/PpPD | ＞4000 | 1600 | 17 |
| **Au(III)** | Polycarbene | 5000 | 2090 | 23 |
| **Au(III)** | H-bond COF | 300 | 1725 | 24 |
| **Au(III)** | Porous porphyrin | ＞3000 | 1970 | 25 |
| **Au(III)** | MOF/PpPDA | 1000 | 934 | 26 |
| **Au(III)** | Amidoxime-MOF | ＞600 | 954 | 27 |
| **Au(III)** | $NH_2$-CTFs | >25 | 909 | 28 |
| **Au(III)** | N-MXene | 1000 | 1198 | 29 |
| **Au(III)** | Py-$MoS_2$ | ＞1700 | 3343 | 30 |
| **Au(III)** | GO/CS sponge | >200 | 3573 | 31 |
| | | >2000 | 7810 | |
| **Au(III)** | S-PAcH | >200 | ~2847 | 32 |
| **Au(III)** | CNT/2H-$MoS_2$ | 1000 | 2495 | 33 |
| **Au(III)** | $TaS_2$ | 10 | 4100 | This Work |
| | | 1 | 3966 | |
| | $TiS_2$ | 100 | 8073 | |
| | | 10 | 8076 | |
| | | 1 | 8084 | |
| **Pd(II)** | CITCF | ~500 | ~929 | 34 |
| **Pd(II)** | POP-o$NH_2$-Py | ~800 | 752 | 35 |
| **Pd(II)** | MXene | ~200 | 184.56 | 36 |
| **Pd(II)** | COF-TzDa | ~482 | 265.4 | 37 |
| **Pd(II)** | MOF-$NH_2$ | ~1600 | 167 | 38 |
| **Pd(II)** | PAN/MOF-$NH_2$ | ＞700 | 165.8 | 39 |
| **Pd(IV)** | Py-$MoS_2$ | ~500 | ~1000 | 30 |
| **Pd(IV)** | Py-$SnS_2$ | >400 | 1103.72 | 15 |
| **Pd(II)** | $TaS_2$ | 10 | 1100 | This Work |
| | | 1 | 1009 | |
| | $TiS_2$ | 100 | 2359 | |
| | | 10 | 2217 | |
| | | 1 | 1892 | |
| **Pt(IV)** | Py-$SnS_2$ | ＞400 | 617.61 | 15 |
| **Pt(IV)** | DG-POP-2 | ＞50 | 388 | 40 |
| **Pt(IV)** | Py-$MoS_2$ | ~500 | ~829 | 30 |
| **Pt(IV)** | PAN/MOF-$NH_2$ | ＞700 | 172.5 | 39 |
| **Pt(II)** | SCU-COF-3 | ＞200 | 168.4 | 41 |
| **Pt(IV)** | Chitosan- l-lysine | ~400 | ~129 | 42 |
| **Pt(II)** | supramolecular polymers | 1-2 | 33 | 43 |
| **Pt(IV)** | S-PAcH | ~200 | ~714 | 32 |
| **Pt(II)** | BIT-POPs | ＞250 | ~360 | 44 |
| **Pt(II)** | COP-180 | ~1000 | ~197 | 25 |
| **Pt(IV)** | $TaS_2$ | 10 | 452 | This Work |
| | | 1 | 439 | |
| | $TiS_2$ | 100 | 1154 | |
| | | 10 | 813 | |
| | | 1 | 323 | |

To analyze the PM extraction difference between $TiS_2$ and $TiS_2$, we have performed the following comparison. The molecular weights (MW) are $MW_{TaS2}$=245 g/mol and $MW_{TiS2}$=112 g/mol, yielding a ratio $MW_{TaS2}$/ $MW_{TiS2}$ =2.2. Comparatively, $Q_e$ for $TiS_2$ was ~8076 mg/g, and for $TaS_2$ was ~4100 mg/g, giving a ratio $Q_e$-$TiS_2$/$Q_e$-$TaS_2$=1.97. The close alignment between these ratios (2.2 vs. 1.97) suggests that molecular weight was a key factor influencing adsorption performance.

Fundamentally, this good match was in good agreement with our mechanism understanding that the electron donation from the sulfur site accounts for the extraction capacity (Fig.4a for $TiS_2$ and Fig. S22 for $TaS_2$). Two TMD nanosheets have similar work functions (Fig. S17), it was again in good agreement with our previous explanation that the energy level of the adsorbent defines its PM adsorption performance.

2.6 Continuous gold extraction using $TiS_2$ aerosol and membrane

For using the $TiS_2$ aerosol as the filter for gold capturing, as shown in Fig. S9, the flattened $TiS_2$ aerosol rapidly captures 300 ppm Au ion (we choose this concentration as it shows a yellowish appearance for the reader to quickly identify the change before and after filtration) by simply and rapid pushing the gold solution through the aerosol using a syringe.

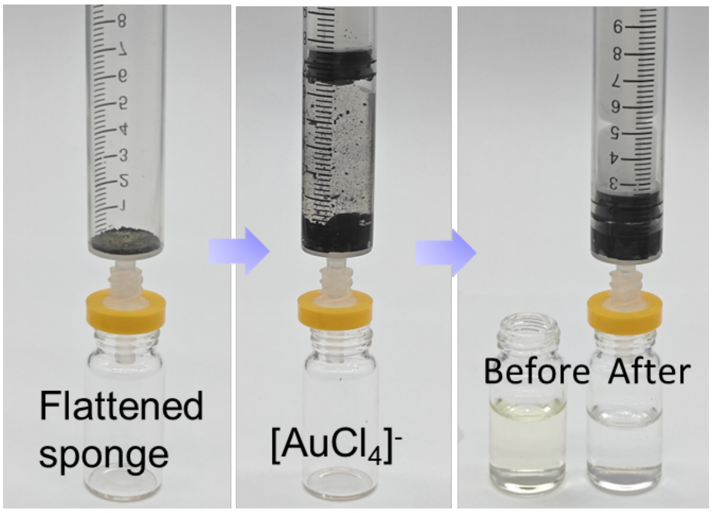


**Fig. S9** Flattened aerosol sponge used for rapid gold capture.

For membrane filtration (Fig. S10a), we used a vacuum filtration setup, where a wet $TiS_2$ membrane was placed between the feed and filtrate parts for gold recovery. During the process, the feed gold solution was suctioned and passed through the membrane, achieving gold recovery. As shown in Fig. S10b, the results showed that a ~1 μm

membrane (effective area ~11.34 $cm^2$) could effectively capture gold from ~1.25 L of 10 ppm gold solution, maintaining ~95% gold recovery efficiency. Note that the permeance increased from an initial ~21 to ~280 $L \cdot m^{-2}h^{-1}bar^{-1}$ over 15 h of filtration. This increase was likely because, during the filtration process, the gold particles grew and the interlayer space of the $TiS_2$ membrane expanded, increasing the permeance. With its capability for continuous capture of PM ions, the $TiS_2$ membrane holds a unique advantage for process scale-up.

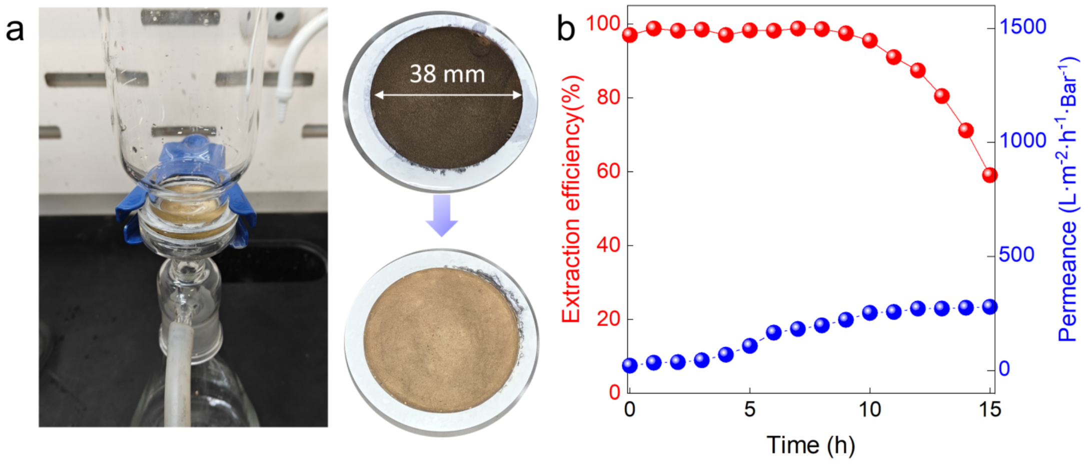


**Fig. S10** Separation of PM adsorbed $TiS_2$ after gold extraction. (a-b) $TiS_2$ membrane-based continuous filtration of gold solution. (a) Filtration setup, the right panel was typical $TiS_2$ membrane appearance before and after the filtration test. (b) The changes in membrane permeance and extraction efficiency with time.

## Supplementary Section 3 | Supplementary evidence for the PM extraction mechanism

3.1 Characterization of $TiS_2$@PM

After PM extraction, the PM-adsorbed $TiS_2$ nanosheets were collected and analyzed using TEM, XPS, and XRD. We first observed the $TiS_2$ nanosheets after 10 min adsorption of $[AuCl_4]^-$, $[PdCl_4]^{2-}$, and $[PtCl_6]^{2-}$, as shown in Fig. S11, the TEM images show that the Au, Pd, and PtS nanoparticles with a diameter ~1-16 nm are densely deposited on the TMD nanosheets, consistent with observed rapid adsorption kinetic.

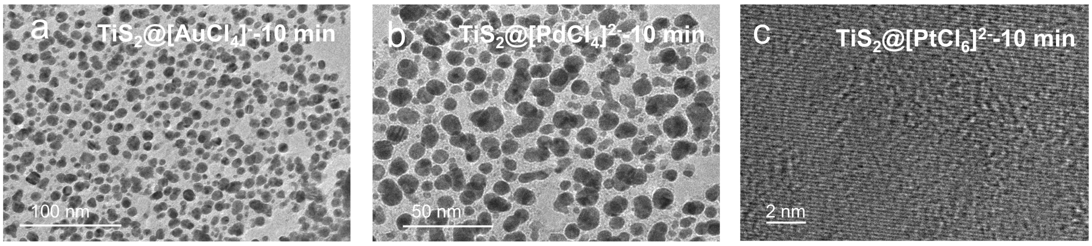


**Fig. S11** TEM images of $TiS_2$ nanosheets for 10 min adsorption of (a) $[AuCl_4]^-$, (b) $[PdCl_4]^{2-}$, and (c) $[PtCl_6]^{2-}$

TEM coupled with energy-dispersive X-ray spectroscopy (EDS) was used to study the distribution of chemical elements in $TiS_2$@PM (Fig. S12). All PM elements were evenly distributed on both the edges and the in-plane surfaces of the $TiS_2$ nanosheets.

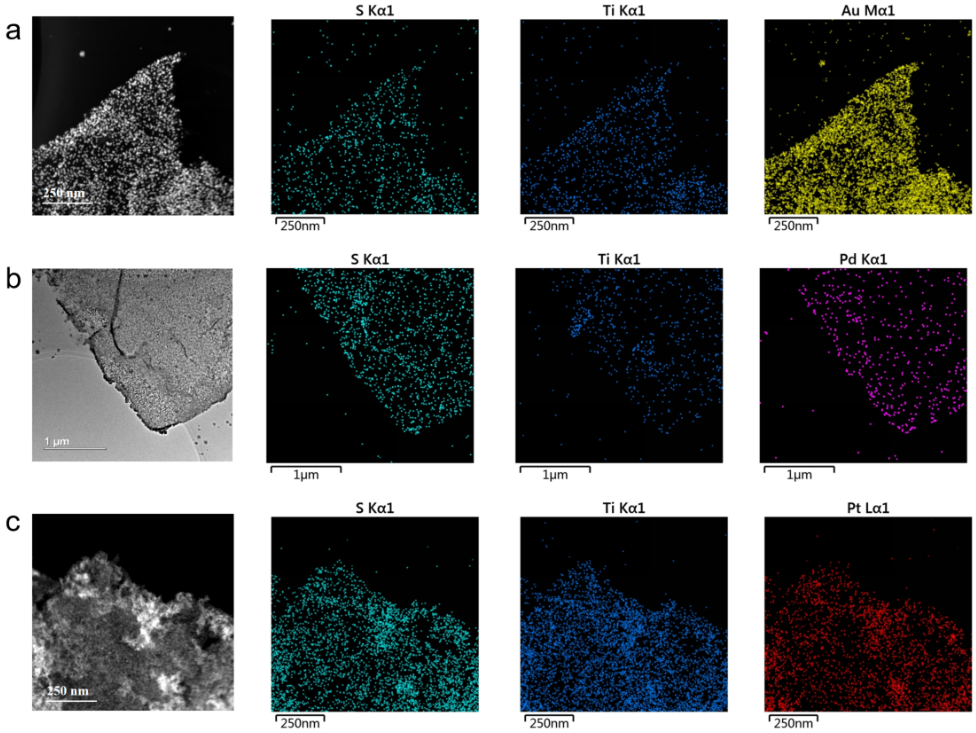


**Fig. S12** TEM-EDS elemental analysis of $TiS_2$ nanosheets after (a) Au, (b) Pd, and (c) Pt adsorption (24h).

To supplement the TEM observation in Fig. 3, Fig. S13 showed the high-resolution TEM images and the corresponding SAED patterns of $TiS_2$@PM, confirming the simultaneous deposition of $Au^0$, $Pd^0$, and PtS nanoparticles.

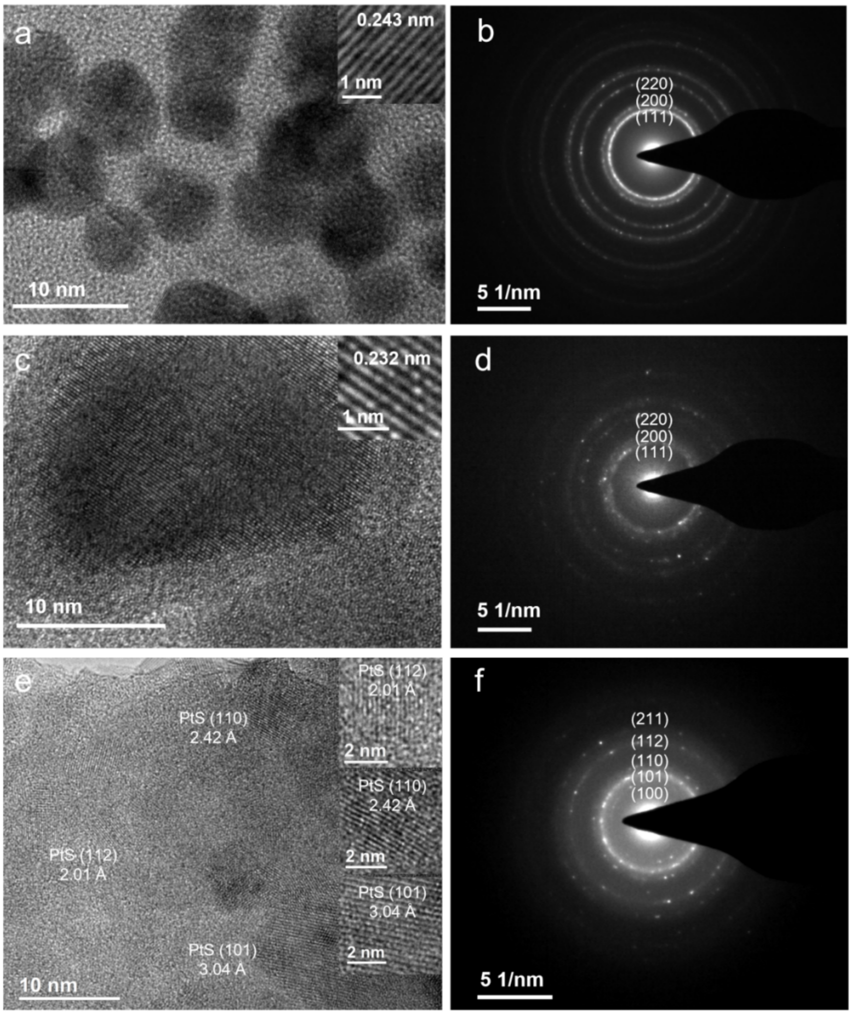


**Fig. S13** Simultaneous deposition of PM-containing nanoparticles. (a) High-resolution image of $TiS_2$@$[AuCl_4]^-$ and (b) corresponding SAED pattern. (c) High-resolution image and (d) corresponding SAED pattern acquired from $TiS_2$@$[PdCl_4]^{2-}$. (e) High-resolution image and (f) corresponding SAED pattern acquired from $TiS_2$@$[PtCl_6]^{2-}$. The corresponding crystalline orientations of Au, Pd, and PtS were labeled in (b), (d), and (f), respectively.

Besides the TEM observation, we also tracked the structural change of individual $TiS_2$ nanosheets deposited on a $Si/SiO_2$ substrate during gold adsorption (10 ppm), the gold ions were excessive with a weight ratio to $TiS_2$ >$10^4$. To facilitate optical microscope observation, we use large $TiS_2$ nanosheets (a few micrometers), they were obtained by redispersing the sediments in water after 3000 rpm centrifugation of the pristine suspension. As shown in Figure S14, after 1 minute, the nanosheet showed a

lighter contrast, likely due to gold deposition and partial sulfur loss. Over 60–120 minutes (near $Q_e$), no further contrast change or dissolution was observed, confirming that while sulfur was oxidized, the $TiS_2$ nanosheet remained structurally intact.

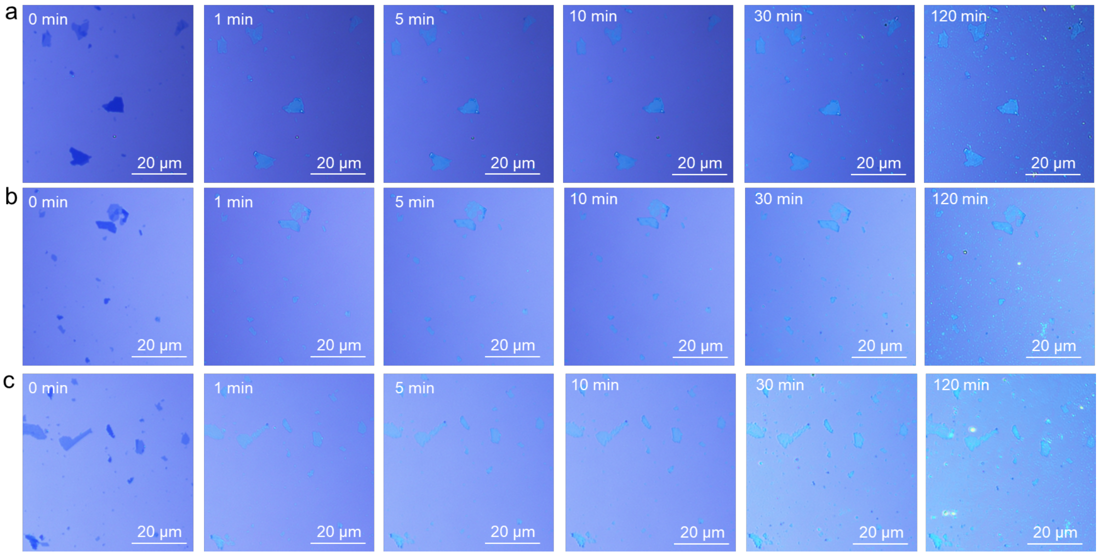


**Fig. S14** Optical images tracking the evolution of $TiS_2$ nanosheets during the gold extraction process.

The structural integrality during adsorption was also supported by the ICP analysis on the Ti concentration after 24 h gold extraction, as shown in Table S3, only < 6wt% of Ti was released to the solution, confirming little structural change and recyclability of Ti.

**Table S3** Comparison of Ti concentration before and after PM extraction

| | **Equivalent Ti concentration based on $TiS_2$ used for extraction (ppm)** | **Ti concentration in solution after extraction (ppm)** |
|---|---|---|
| Au | 4.28±0.05 | 0.057±0.008 |
| Pd | 14.25±0.08 | 0.485±0.067 |
| Pt | 21.38±0.13 | 0.082±0.005 |

We also used X-ray photoelectron spectroscopy (XPS) to examine the chemical composition of $TiS_2$@PM precipitates. The wide-range XPS spectra of $TiS_2$ after absorption showed the existence of O1s, Ti2p, S2p, Pd3d, Pt4f, and Au4f peaks (Fig. S15). Notably, XPS of $TiS_2$@$[AuCl_4]^-$ showed a weak sulfur peak, which was consistent with $S^{2-}$ being oxidized to soluble sulfate species.

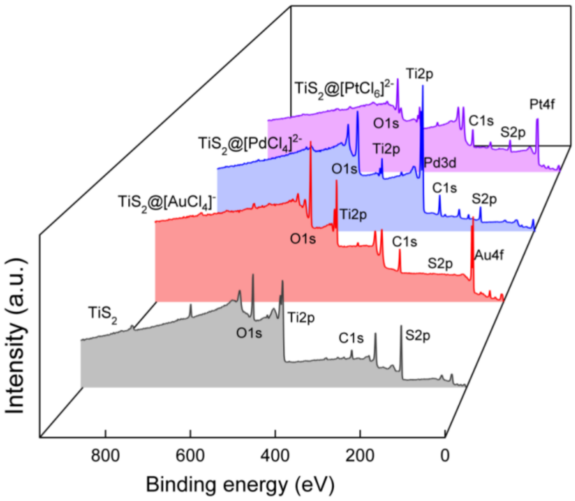


**Fig. S15** Wide-range XPS spectra of $TiS_2$ nanosheets and $TiS_2$@PM after 24 hours of adsorption.

Not only $[AuCl_4]^-$, $[PdCl_4]^{2-}$, and $[PtCl_6]^{2-}$, $TiS_2$ nanosheets were efficient for other Au-, Pd-, Pt- and Ag-containing ions with a reduction potential higher than +0.59 V, in addition, to supplement the $Q_e$ shown in Fig. 3 of the main text, we had provided further XRD spectra of $TiS_2$ after its extraction of $[AuBr_4]^-$, $Ag^+$($AgNO_3$), $[AuI_4]^-$, $[RhCl_6]^{3-}$ and $[Au(S_2O_3)_2]^{3-}$. As shown in Fig. S16, the diffraction peaks assigned to metallic Au, and Ag appeared in the XRD patterns of $TiS_2$@$[AuBr_4]^-$, $TiS_2$@$[AuI_4]^-$, and $TiS_2$@$Ag^+$, suggesting a reductive extraction, consistent with the observed high $Q_e$ shown in Fig. 3, but the diffraction peaks for metallic gold were absent for $[Au(S_2O_3)_2]^{3-}$ for which $TiS_2$ had a negligible $Q_e$, similar to $[RhCl_6]^{3-}$.

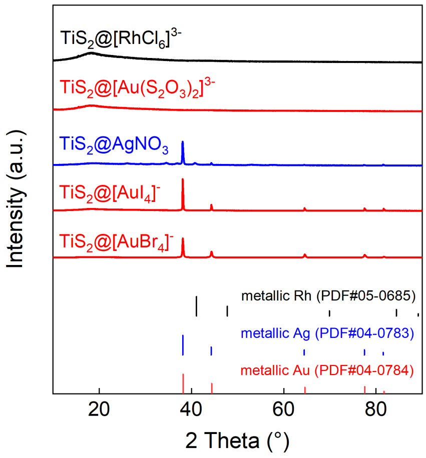


**Fig. S16** XRD patterns of $TiS_2$@$[AuBr_4]^-$, $TiS_2$@$[AuI_4]^-$, $TiS_2$@$[Au(S_2O_3)_2]^{3-}$, $TaS_2$@$AgNO_3$, and $TiS_2$@$[RhCl_6]^{3-}$. The diffraction peak positions of metallic Rh, Ag, and Au from the standard PDF card were indicated at the bottom.

3.2 Work functions of $TiS_2$ and $TiS_2$ nanosheets

The work function (Φ) of semimetallic $TiS_2$ and $TaS_2$ nanosheets was a direct indicator of their reduction potential; we, therefore, used ultraviolet photoelectron spectroscopy (UPS) to measure their Φ.

Φ was the minimum energy required to move an electron from inside a solid to its surface, and was defined as the difference between the vacuum level ($E_{vac}$) and the Fermi level ($E_F$), which could be expressed as follows:

$$\Phi = E_{vac} - E_F \qquad \text{Eq. (8)}$$

where $E_F$ was defined as the highest energy level occupied by electrons at T = 0 K., and $E_{vac}$ was the energy at which an electron was completely free and not influenced by the nucleus and was generally 0 eV.

Furthermore, because the difference between the vacuum level and the standard hydrogen electrode potential was 4.44 eV, the relationship between Φ and the reduction potential (versus Standard Hydrogen Electrode (vs SHE)) of a given material/ion was written as follow[45]:

$$E\ (vs.\text{SHE}) = \Phi/e - 4.44\ \text{V} \qquad \text{Eq. (9)}$$

Using Eq. (9), the work functions of $TiS_2$ and $TaS_2$ nanosheets can be translated into their reduction potentials, explaining their reductive adsorption to the PM ions.

Fig. S17 showed the UPS spectra of $TiS_2$ and $TaS_2$ that we used to determine Φ. According to Eq. (10),

$$\Phi = h\upsilon - (E_{cutoff} - E_F) \qquad \text{Eq. (10)}$$

where $E_{cutoff}$ was the inelastic secondary electron cutoff, $E_F$ was the Fermi level edge[46], and the photon energy (hυ) of the excitation light used for our measurement was 21.22 eV. $E_{cutoff}$ values of $TiS_2$ and $TaS_2$ were respectively determined to be 17.27 and 17.33 eV, and the $E_F$ of $TiS_2$ and $TaS_2$ was ~0, yielding the work functions for $TiS_2$ and $TaS_2$ of 3.89 eV and 3.95 eV, respectively. Using Eq. (9), the reduction potentials of $TiS_2$ and $TaS_2$ were respectively -0.55 V and -0.49 V (vs. SHE)[45, 47, 48],

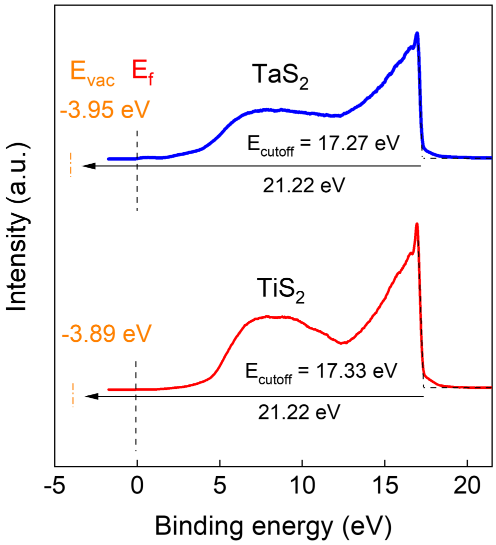


**Fig. S17** UPS spectra for work function measurements.

To supplement Fig. 3f and g, Fig. S18 showed the reduction potential of the ions used in this study versus the calculated equivalent reduction potential of $TiS_2$ and $TaS_2$, which clearly showed that an energy difference of ~1 eV was required for the reductive extraction by $TiS_2$ and $TaS_2$[47, 48], and such a difference could be due to the energy required for ion diffusion, ion desolvation, and the crystal nucleation and growth of Au, Pd, and PtS.

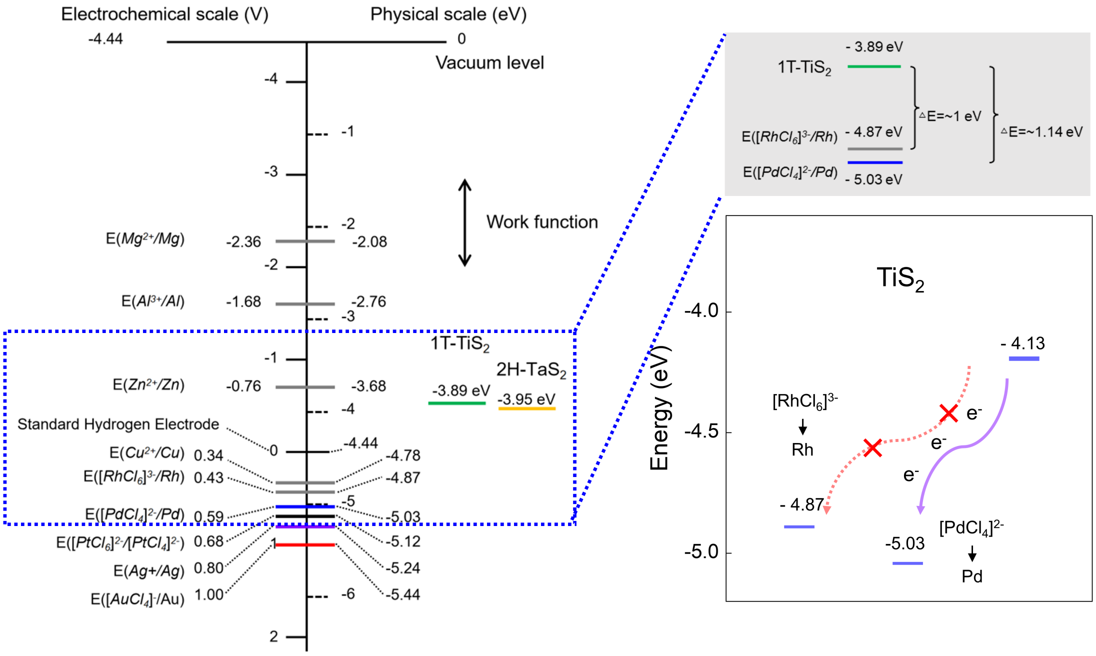


**Fig. S18** Comparison of reduction potentials of the ions studied and the work functions of $TiS_2$ and $TaS_2$ nanosheets. Right panels show the Band alignment diagram for semimetallic $TiS_2$ nanosheets, $[PdCl_4]^{2-}/Pd^0$ and $[RhCl_6]^{3-}/Rh^0$.

3.3 Gold extraction capacities of the unexfoliated TMD and exfoliated ones

To study the influence of surface area on $Q_e$, we measured the $Q_e$ ($C_0$ =100 ppm) for $[AuCl_4]^-$ of unexfoliated TMDs using an identical procedure used for the exfoliated ones. As shown in Fig. S19, the $Q_e$ values of unexfoliated $TiS_2$ and $TaS_2$ were <1000 mg/g, significantly lower than for the exfoliated nanosheets, suggesting the large surface area of the exfoliated ones provides more PM adsorption sites, giving a high $Q_e$.

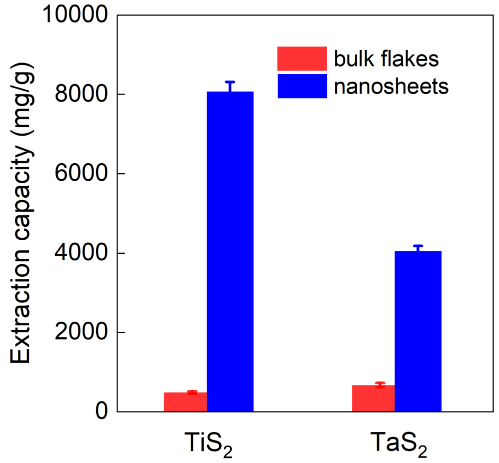


**Fig. S19** Extraction capacity of $TiS_2$ and $TaS_2$ nanosheets compared with flakes.

3.4 XPS analysis of TMD and the byproduct salt after PM extraction

Fig. S20 compared the XPS peaks of Ti and S before and after gold extraction. Compared with the pristine $TiS_2$, both Ti2p and S2p peaks shifted to a higher binding energy, suggesting the proposed electron donation. The deconvoluted Ti2p peaks showed that the Ti-S bond had changed to the Ti-O bond after gold extraction.

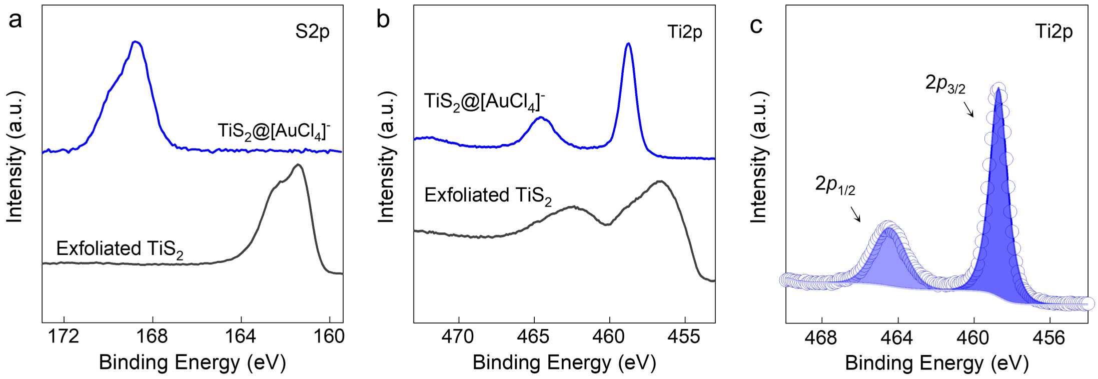


**Fig. S20** XPS peaks of Ti and S before and after gold extraction. (a) XPS S2p spectra of dissolved salt collected after PM adsorption compared with pristine $TiS_2$. (b) XPS Ti2p spectra of $TiS_2@[AuCl_4]^-$ precipitates compared with pristine $TiS_2$. (c) Deconvoluted XPS Ti2p spectra of $TiS_2@[AuCl_4]^-$ precipitates.

Further XPS analysis was performed on the $TiS_2$@PM with adsorption times of 1, 5, and 10 minutes, and also supported that sulfur was the primary electron-donating site. As a 10-minute adsorption of gold nearly reached its adsorption equilibrium, we focused on the structural evolution of $TiS_2$ for 1 min, 5 min, and 10 min of gold adsorption. As shown in Fig. S21, for $[AuCl_4]^-$, after 1 min of adsorption, 93% of $[AuCl_4]^-$ was reduced to $Au^0$, with the rest being $Au^{1+}$, consistent with the observed rapid and reductive adsorption. The XPS spectra of S2p showed that S remained Ti-S bonds similar to the pristine $TiS_2$ (Fig.S20), but we observed atomic ratio(S: Ti) changed from pristine ratio of ~2 to 1.22, 0.74 and 0.63 for 1, 5 and 10 min adsorption, indicating sulfur loss, this aligns with our mechanism explanation that, during adsorption, Ti showed negligible leaching in water, but $S^{2-} \rightarrow S^{6+}$, these oxidized sulfate were released in water while the adsorbent remained structural integrity but in its oxidized form.

These structural evolutions of $TiS_2$ provided another piece of evidence on the adsorption mechanism. Our mechanism suggested that sulfur donates electrons for PM recovery—sulfur was then oxidized into sulfate and released into water. Here, it should be noted that (1) the $TiS_2$ nanosheets with sulfur loss and partial oxidation retained their structural integrity, a critical quality for their use as an adsorbent rather than a chemical regent. Such structural integrity during oxidation was also in good agreement with two recent publications that report the partial oxidation of TMD nanosheets remains solid 2D nanosheets[49, 50]. (2) Our XPS analysis did not show other sulfur species in the solution; thus, all $S^{2-}$ was converted to $SO_4^{2-}$.

If our mechanism stands, then the XPS measured sulfur loss from $TiS_2$ nanosheet was a process in which each sulfur donated 8 electrons and became sulfate, being released into water. This allowed us to calculate the $Q_t$ (adsorbed gold at a given adsorption time). For example, for 1 mole $TiS_2$, at 1 min, the S: Ti ratio decreased from 2 to 1.22. That is,

(a) The mole number of sulfur that donated its 8 electrons was 2-1.22=0.78 mole.

(b) The mole number of electrons donated from sulfur to $[AuCl_4]^-$: 0.78*8= 6.24 moles.

(c) As $[AuCl_4]^- \rightarrow Au^0$ required 3 electrons (XPS results showed that even at 1 min, >91% $[AuCl_4]^- \rightarrow Au^0$ occurred, with the rest being $Au^+$), then 6.24 moles of electrons donated from sulfur would yield 6.24/3 = ~2.08 moles of $Au^0$.

(d) That is, based on XPS results, if our proposed mechanism stands, at 1 min adsorption, 1 mole $TiS_2$ should reductively adsorb 2.2 mole $Au^0$, yielding a $Q_t$= mass of adsorbed gold/mass of used $TiS_2$ = (2.08*197)/(1*112) = 3.66 g/g. This

was in good agreement with the measured $Q_t$, which was 3.54 g/g (Fig. S5).

(e) Following the same method, we calculated the $Q_t$ at 5 and 10 min to be 5.79 g/g and 6.30 g/g; again, we found good agreement between the calculated $Q_t$ and the experimentally measured $Q_t$ (5.47 g/g and 6.42 g/g for 5 and 10 min adsorption).

This unambiguously supported the mechanism that the reported ultrahigh adsorption was reductive adsorption, and sulfur was the major electron donor, accounting for such high extraction capacity.

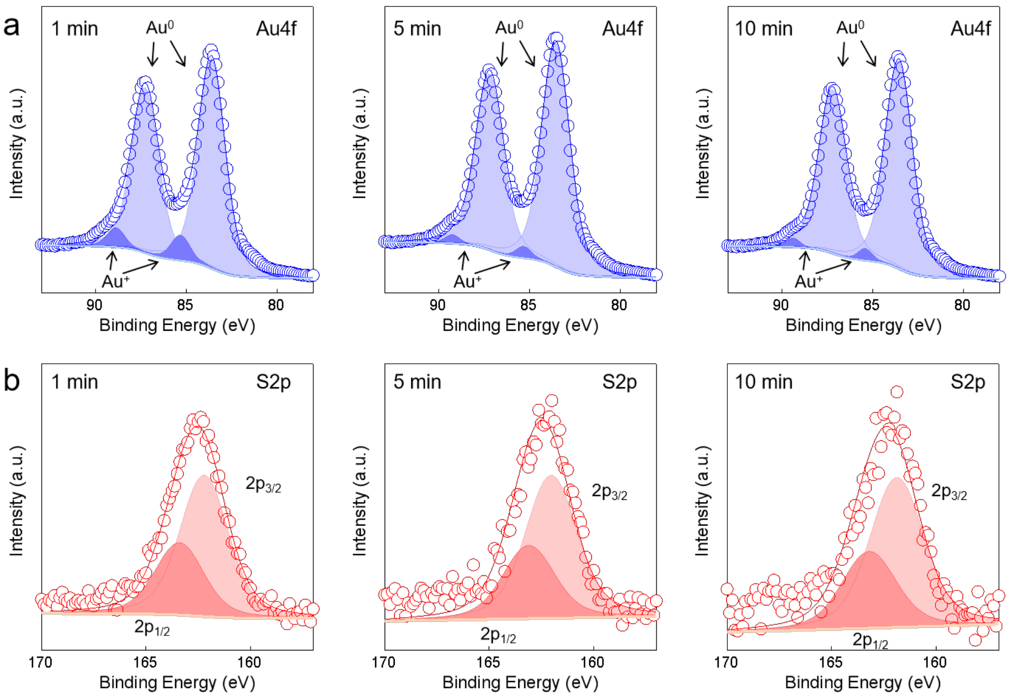


**Fig. S21** Structural evolution of $TiS_2$ for 1 min, 5 min, and 10 min gold adsorption. Evolution of (a) deconvoluted XPS Au4f spectra and (b) deconvoluted XPS S2p spectra.

Not only the sulfur of $TiS_2$ (Fig. 4a) but also that of $TaS_2$ was the primary electron donation site. As shown in Fig. S22, we first analyzed the sulfur dissolved during the gold extraction by $TaS_2$, the peaks at 168.6 eV and 169.8 eV were assigned to sulfate species, similar to Fig. 4a. Furthermore, Ta4f of $TaS_2$ after gold extraction showed two peaks at 26.6 and 28.5 eV, both assigned to $Ta^{5+}$ (Fig. S22b)[51, 52], suggesting that $Ta^{4+}$ was oxidized to $Ta^{5+}$ during the reductive extraction.

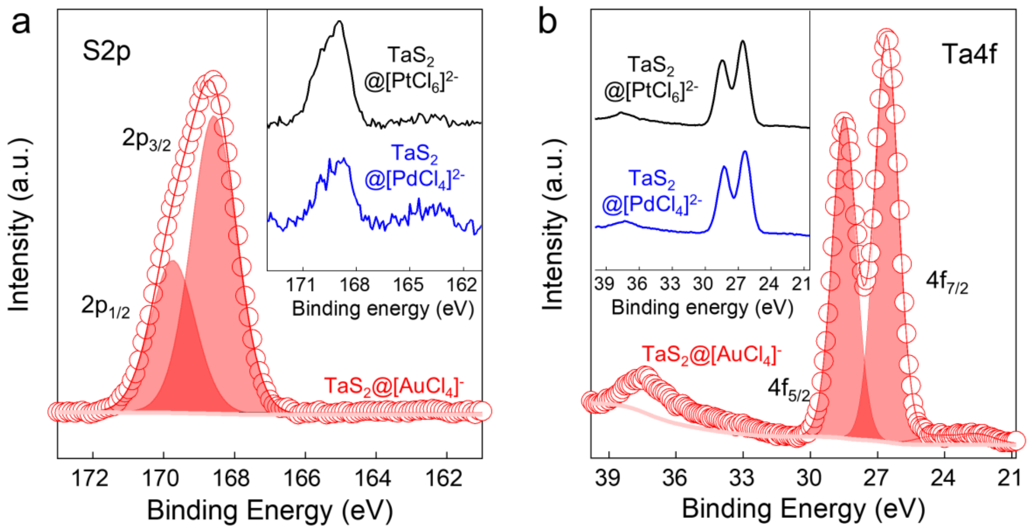


**Fig. S22** XPS analysis for the transformation of sulfur for $TaS_2$ after PM adsorption. (a) Deconvoluted S2p peaks of dissolved compounds collected after PM adsorption by $TaS_2$. The red line represents $TaS_2$@$[AuCl_4]^-$. Inset were the XPS S2p spectra of $TaS_2$@$[PdCl_4]^{2-}$ (blue line) and $TaS_2$@$[PtCl_6]^{2-}$ (black line). (b) Deconvoluted Ta4f peaks for the $TaS_2$@PM precipitate after adsorption. The red line represents $TaS_2$@$[AuCl_4]^-$. Inset were the XPS S2p spectra of $TaS_2$@$[PdCl_4]^{2-}$ (blue) and $TaS_2$@$[PtCl_6]^{2-}$ (black).

3.5 Calculation of the electron donation number during reductive extraction

The number of donated electrons from each $TiS_2$ molecule to $[AuCl_4]^-$ was calculated using Eq. (11).

$$N_e = \frac{Mr_{TiS_2} \times Q_e}{Mr_{Au}} \times n \qquad \text{Eq. (11)}$$

where $N_e$ was the electron donation numbers per TMD molecule. $M_{TiS_2}$ and $M_{Au}$ were the molar masses of $TiS_2$ and Au, respectively. $Q_e$ was the extraction capacity of $TiS_2$ for $[AuCl_4]^-$. $n$ was 3 as three electrons were required for the reduction of $[AuCl_4]^-$ to $Au^0$. For a $Q_e$ of 8076 mg/g for $[AuCl_4]^-$.

The rationale behind Eq.(11) could be understood as follows,

**Step 1:** The XPS results showed that 99% of $[AuCl_4]^-$ was reduced to $Au^0$, requiring 3 electrons per ion. For simplicity, we assume 100% reduction.

**Step 2:** Our extraction capacity was ~8 g/g, meaning 1 g $TiS_2$ (112 g/mol) can reductively adsorb 8 g $Au^0$ (~197 g/mol). Converting to moles, 1/112 moles of $TiS_2$ adsorb 8/197 moles of $[AuCl_4]^-$ and reduce them to $Au^0$.

**Step 3:** thus, 1/112 moles of $TiS_2$ donates 3×(8/197) moles of electrons. Scaling to 1 mole of $TiS_2$ yielded ~13.7 moles of electrons (3 × 8×112/197 ≈ 13.7). We report "no

fewer than 13 electrons" as a conservative estimate, with ~13.7 as the calculated average.

3.6 The role of water in the gold extraction process by $TiS_2$ nanosheets

The critical role of water was probed by monitoring the pH change during gold extraction, as the water dissociation released $H^+$, decreasing the pH. Indeed, as shown in Fig.S23, the pH decreased from 3.96 to 3.77 during the 24-hour adsorption process, confirming the dissociation of water.

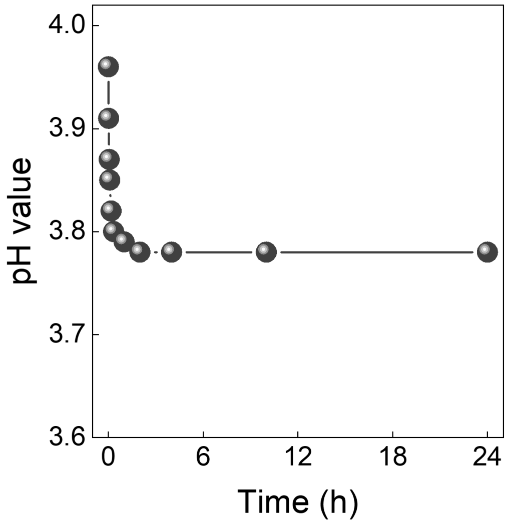


**Fig. S23** pH value of the $KAuCl_4$ aqueous solution ($C_0$ =10 ppm) after adding $TiS_2$ nanosheets.

Next, the solvent used for gold extraction was replaced by acetone. To determine the $[AuCl_4]^-$ concentration in acetone, we measured the UV-Vis spectra of a series of acetone solutions with $[AuCl_4]^-$ concentrations from 0 ppm to 100 ppm, and found a good linearity ($R^2$=0.999) between concentration and absorption at 340 nm (Fig. S24a). We, therefore, used a $C_0$ of 100 ppm to study the influence of the organic solvent on $Q_e$, and found its $Q_e$ decreased to 1180 mg/g. Adding water to the acetone (water volume ratio of 30%), again, we used UV-Vis spectra to determine the ion concentration and found linearity between the concentration and the adsorption intensity (ABS) at 340 nm (Fig. S24b), with this linear relationship ($R^2$=0.999), we determined that $Q_e$ after adding 30 vol% water to the acetone increased to 5217 mg/g.

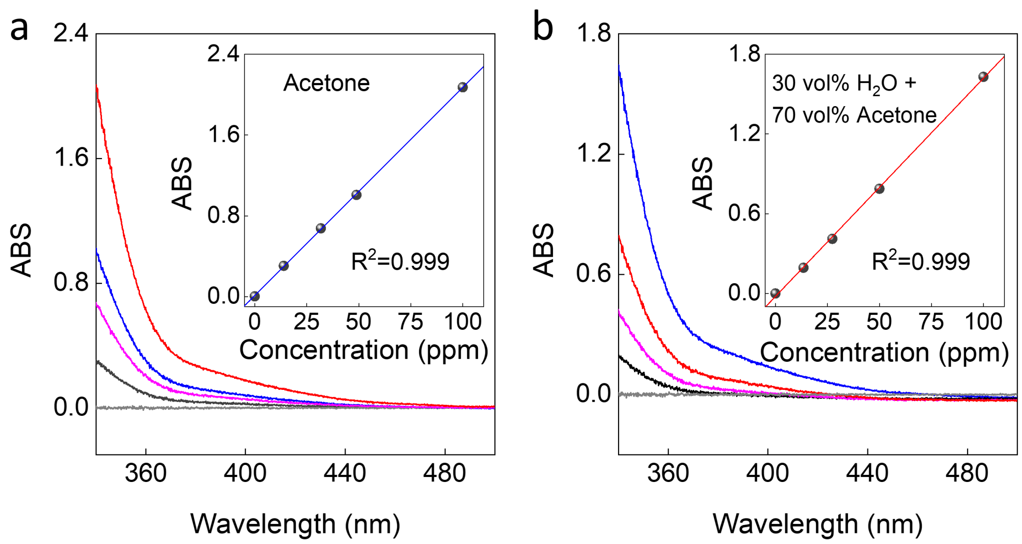


**Fig. S24** Evolution of the UV−vis spectra for different solutions with different $KAuCl_4$ concentrations. UV−vis spectra of (a) acetone and (b) 30 vol%-water-70 vol%-acetone solution of $KAuCl_4$. Insets were linear relationships between the absorbance at λ = 340 and the corresponding $KAuCl_4$ concentration.

3.7 DFT calculations

To understand the role of water in the extraction process, spin-polarized density functional theory (DFT) calculations were conducted as implemented in the Vienna Ab Initio Simulation Package (VASP). The interactions between ions and electrons were described using projector-augmented wave (PAW) pseudo-potentials. The exchange-correlation effects were treated with the Perdew-Burke-Ernzerhof (PBE) functional within the framework of the generalized gradient approximation (GGA).

A monolayer slab model of 1T-$TiS_2$ was constructed to represent the dechlorination reduction process of $[AuCl_4]^-$ by $TiS_2$ in an aqueous solution. For the structural optimization of 1T-$TiS_2$ crystal cells, a plane-wave energy cutoff of 600 eV was used, along with Gaussian smearing of 0.05 eV. A gamma-centered Monkhorst-Pack of 9×9×5 k-point grids was used for 1T-$TiS_2$. To simulate the two-dimensional interface, periodic structures of 8×8×1 supercells of 1T-$TiS_2$ were constructed with a vacuum thickness of 15 Å.

For the calculations, a reduced plane-wave energy cutoff of 450 eV was used, along with Gaussian smearing and a 2×2×1 gamma-centered Monkhorst-Pack k-point grid. Electronic self-consistency was achieved with an energy convergence criterion of $10^{-5}$ eV, and structural relaxations were performed until the forces on all atoms were below

0.02 eV/Å for all calculations. In addition, the implicit solvent model was used to compute the energy diagram of the dechlorination reduction process of $[AuCl_4]^-$ and 1T-$TiS_2$ in aqueous solution.

## Supplementary Section 4 | PM Recovery from real-world waste streams

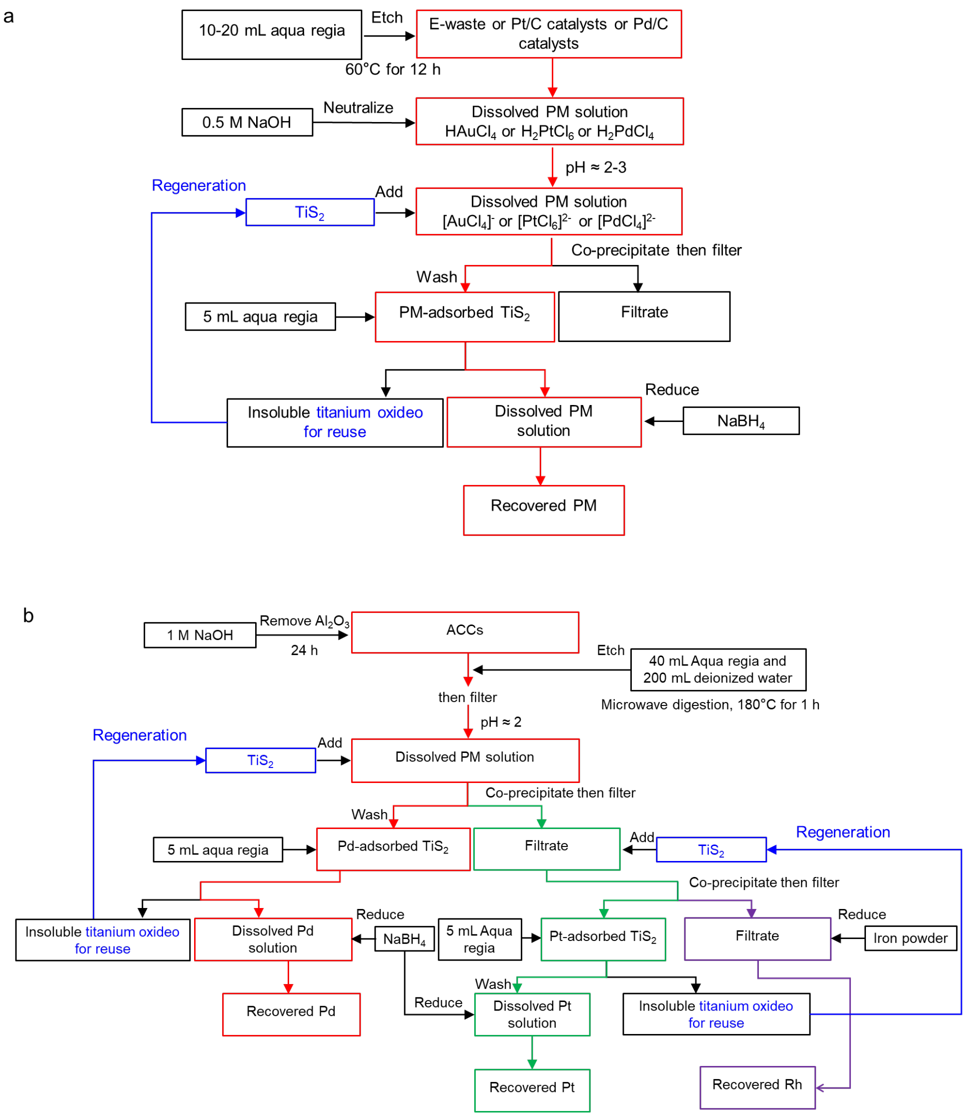


**Fig. S25** Flowchart for PM recycling from its waste streams.

As shown in Fig. S25, for Au recovery from electronic waste (e-waste), five pieces of scrap computer central processing unit (CPU) boards were digested using 20 mL aqua regia at 60 °C for 12 hours. Subsequently, the solid waste was removed by filtering the digested mixture through a 0.22 µm pore size polyethersulfone (PES) membrane. The pH of the filtered leachate was then adjusted using 0.5 M NaOH to give a pH of ~2, resulting in an Au ion concentration of ~16.3 ppm. Subsequently, $TiS_2$ nanosheets (~3 mg) were added to the 100 mL leachate with stirring for 24 hours. Finally, the

resulting mixture was filtered through a 0.22 μm membrane to obtain Au-adsorbed $TiS_2$ (>95% could be separated) and the filtrate. The removal efficiency of the filtrate was determined by ICP-OES using Eq. (2) in the supplementary section 2. The obtained Au-adsorbed $TiS_2$ was washed with deionized water and dissolved in 5 mL of aqua regia for 12 hours, yielding a concentrated Au solution and insoluble Ti-containing precipitates. We used scanning electron microscopy (SEM, Hitachi SU8010) -EDS to quantify the element composition of these precipitates and found that Ti (61 wt%), O (33 wt%), and other elements (~6 wt%, such as C) were present in the sample (Fig. S26b). The Au ions were reduced with $NaBH_4$ to obtain a metallic gold nugget. EDS analysis confirmed that the sample contained over 97 wt% Au (Fig. S26a), with ~3 wt% of carbon and oxygen elements possibly from the environment.

For Pt recovery from scrap catalysts, the 0.2 g fuel cell catalyst (Pt/C, obtained from Sinerosz) was first digested using 20 mL aqua regia with stirring at 60°C for 12 hours and then filtered through a 0.22 μm membrane to obtain a Pt-containing leachate. The pH of the leachate was adjusted with 0.5 M NaOH to give a pH of ~3, yielding a Pt concentration of 39.6 ppm. Next, 15 mg of $TiS_2$ was added into a 50 mL leachate with stirring for 24 hours. Subsequently, the resulting mixture was filtered to separate Pt-adsorbed $TiS_2$ and the filtrate. The removal efficiency of the filtrate was determined by ICP-OES. Finally, we used the same chemical stripping and reduction method for Au recycled from the e-wastes to separate Pt from $TiS_2$. EDS analysis (Fig. S26c) showed that the purity of Pt exceeds 97 wt%, with ~3 wt% of C and O present.

For Pd recovery from scrap catalysts, 0.1 g Pd/C catalyst (purchased from Alibaba) was soaked into 10 mL aqua regia for 12 hours, stirring at 60°C, and then filtered to obtain a leachate. The pH of the leachate was adjusted with 0.5 M NaOH to give a pH of ~2, yielding a Pd concentration of ~ 43 ppm. 5 mg of $TiS_2$ was then added to 50 mL of leachate with stirring for 24 hours and further filtered to obtain Pd-adsorbed $TiS_2$ and filtrate, resulting in a Pd concentration reduced to 0.141 ppm and 99% recovery of the Pd. Finally, we used the same chemical stripping and reduction method for Au recycled from the e-wastes, to separate the Pd from $TiS_2$. EDS analysis (Fig. S26d) showed the purity of Pd was > 97 wt%, with ~3 wt% of C and O elements.

For the recycling of platinum and palladium from spent automotive catalytic converters (ACCs), we followed previous reports[53, 54], The spent ACCs (120 g, purchased from Alibaba) were firstly pulverized and soaked in 500 mL of a 1 M NaOH aqueous solution for 24 h to remove $Al_2O_3$. The solid powder was collected by filtration and then digested using 40 mL of aqua regia diluted with 200 mL of deionized water. The mixture was then heated (1500 W, 1 hour) at 180 °C using a microwave digester instrument (Honeycomb XH-800X) to dissolve the PM, after which the mixture was filtered, and the filtrate was the leachate of PM ions (pH ~2). Subsequently, following the process flow depicted in Fig. 5d, we added the first $TiS_2$ nanosheets (20 mg) to the 200 mL leachate to recycle the Pd ions for 10 minutes. The $TiS_2$ with adsorbed Pd was then separated from the leachate by filtration, and a second batch of $TiS_2$ nanosheets (20 mg) was added to the resultant leachate to extract Pt for 36 hours. The $TiS_2$ nanosheets with adsorbed Pt were separated from the leachate, leaving the leachate without or with little Pd and Pt, but ~18 ppm of Rh ions. We added iron powder (100 mg) to this leachate, and recycled Rh was obtained in the form of precipitates. For the separation of Pt and Pd from $TiS_2$, we used the same chemical stripping and reduction method for Au recycled from the e-wastes. The purity of Pd after the first-step purification step was ~88 wt% (with ~11 wt% Pt), while the Pt purity after the second step was ~98 wt% (Fig. S26e and Fig. S26f).

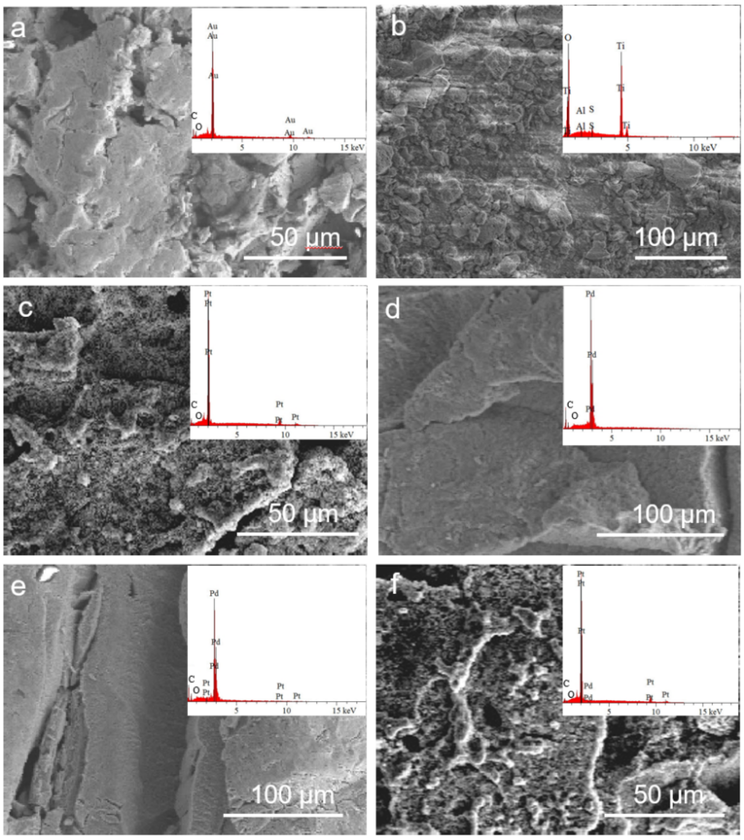

**Fig. S26** SEM-EDS analysis of PM nuggets and Ti-containing precipitates from their recycling process. SEM images of (a) Gold nugget and (b) Ti-containing precipitates from CPU recycling. SEM images of (c) Pt nugget and (d) Pd nugget from Pt/C and Pd/C recycling, respectively. SEM images of (e) Pd nugget and (f) Pt nugget from ACCs recycling. Insets were their corresponding EDS energy spectra.

**Supplementary Section 5 | Adsorbent regeneration after PM extraction**

After desorption, the spent $TiS_2$ adsorbent was treated with flash heating under $CS_2$ flow (Fig. S27a). The flash heating setup (SA-D70260AR, Shenzhen Zhongke Jingyan Technology Co., Ltd.) operated at 640 W input power (32 V, 20 A), reaching ~973 K. Six consecutive flash heating cycles (10 seconds per cycle) were performed. XRD analysis of the resulting products (Fig. S27c) confirmed successful regeneration, showing patterns consistent with crystalline $TiS_2$ (PDF #65-3372). Subsequently, the regenerated $TiS_2$ (Fig. S27b) achieved a gold extraction capacity of 6700 mg/g (Fig. S27d), suggesting a true reuse of $TiS_2$ flakes for PM recovery.

Furthermore, we calculated the energy cost of the flash heating regeneration process, yielding an energy consumption of 38.4 kJ. the energy consumption was decreased to ~0.22 % of that for conventional furnace annealing (~973 K, 4 h)[55], and was only ~0.92 %

compared to the overall energy consumption for exfoliation and PM adsorption by $TiS_2$.

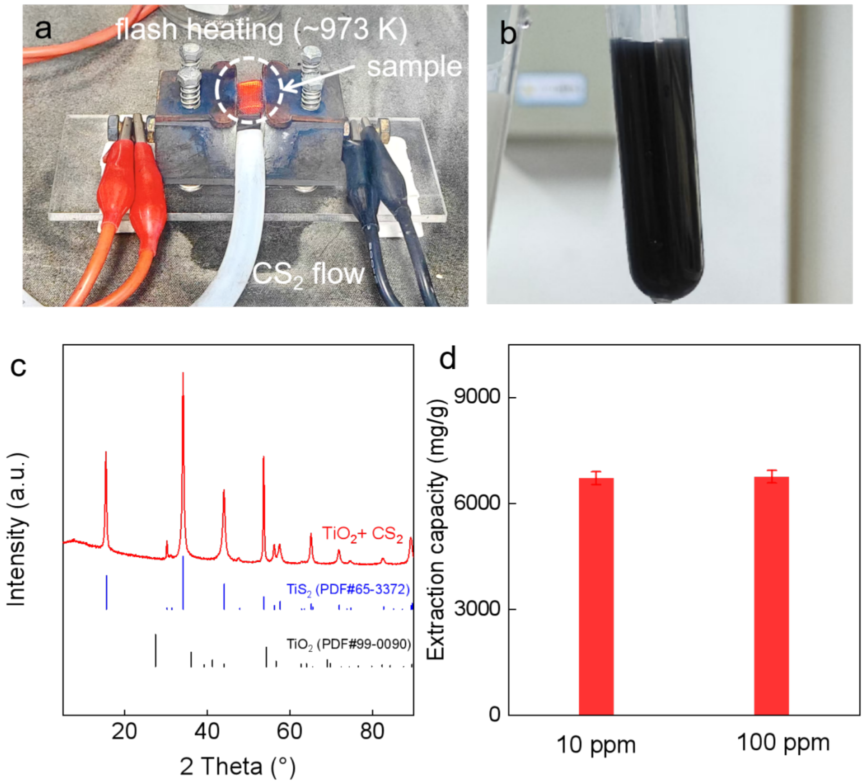


**Fig. S27** (a) Photograph of flash heating setup. (b) Photograph of the regenerated $TiS_2$ dispersion. (c) XRD spectra of regenerated $TiS_2$, and the PDF card data for $TiS_2$ and $TiO_2$ are drawn for comparison. (d) Extraction capacity of regenerated $TiS_2$ for 10 and 100 ppm gold solution.

Finally, we evaluated the environmental impact and carbon emission of the semimetallic TMD for PM recovery. We chose activated carbon (AC) as a reference[56], as it was a widely used adsorbent for gold recovery, and considered the exfoliation of $TiS_2$ and the adsorption process for such evaluation.

For environmental impact, exfoliation of $TiS_2$ uses lithium salt; the subsequent wash of intercalated $TiS_2$ recovers nearly 90% lithium salt, with the rest being diluted as $Li^+$ and $OH^-$(~0.02 M in 200 mL water), which can be neutralized. Therefore, its environmental impact was low. For gold recovery, $TiS_2$ had a high extraction capacity (~8076 mg/g), but AC showed an extraction capacity of ~66 mg/g[51]. Thus, recovering 1 g of gold requires ~0.12 mg of $TiS_2$ versus ~15 mg of AC, significantly reducing material use and associated waste. The adsorption process for both materials used similar aqueous conditions, suggesting comparable process-related impacts, but $TiS_2$'s higher efficiency minimizes environmental footprint.

For carbon emission, as the exfoliation and adsorption do not directly produce $CO_2$ or other greenhouse gas, we calculated the energy cost for adsorption of 1g of gold and compared it with AC. As shown in Table S4, including exfoliation, adsorption-associated energy consumption, and a newly developed flash-heating regeneration method, $TiS_2$ required ~ 516 kJ/g of gold, 122 times lower than AC (~63,127 kJ/g), thanks to a high extraction capacity of $TiS_2$. Considering only 38.4 kJ was required for $TiS_2$ regeneration while thermal activation was generally needed for AC, an even higher energy saving (lower carbon emission) should be expected when considering adsorbent reuse.

Table S4 Energy consumption of major steps of the proposed adsorption process

| Item | Calculation Basis | Energy consumption |
|---|---|---|
| $TiS_2$ Nanosheets | Sonication:120 W for 30 min;<br>Centrifugation: 700 W for 10 min | 636 kJ |
| Adsorption | 20 W for 24 h | 1,728 kJ |
| Aqua Regia Leaching | 20 W for 12 h (CPU leaching)<br>+12 h (gold stripping) | 1,728 kJ |
| $NaBH_4$ Reduction | 20 W for 30 min | 36 kJ |
| $TiS_2$ regeneration | 640 W for 60s | 38.4 kJ |
| Total | | 4,166.4 kJ |

## References


1. Deng, F. et al. Regulating the Electrical and Mechanical Properties of $TaS_2$ Films via van der Waals and Electrostatic Interaction for High Performance Electromagnetic Interference Shielding. *Nano-Micro Letters* **15**, 106 (2023).
2. Li, Y. et al. Making Large-Area Titanium Disulfide Films at Reduced Temperature by Balancing the Kinetics of Sulfurization and Roughening. *Advanced Functional Materials* **30**, 2003617 (2020).
3. Yao, T. et al. Encapsulation of Titanium Disulfide into MOF-Derived N,S-Doped Carbon Nanotablets Toward Suppressed Shuttle Effect and Enhanced Sodium Storage Performance. *Small* **20**, 2311126 (2024).
4. Wang, S. et al. Organic covalent modification to improve thermoelectric properties of $TaS_2$. *Nature Communications* **13**, 4401 (2022).
5. Tang, J. et al. MXene derived $TiS_2$ nanosheets for high-rate and long-life sodium-ion capacitors. *Energy Storage Materials* **26**, 550-559 (2020).
6. Telkhozhayeva, M. et al. 2D $TiS_2$ flakes for tetracycline hydrochloride photodegradation under solar light. *Applied Catalysis B: Environmental* **318**, 121872 (2022).
7. Peng, J. et al. Very Large-Sized Transition Metal Dichalcogenides Monolayers from Fast Exfoliation by Manual Shaking. *Journal of the American Chemical Society* **139**, 9019-9025 (2017).
8. Wu, J. et al. Acid-Assisted Exfoliation toward Metallic Sub-nanopore $TaS_2$ Monolayer with High Volumetric Capacitance. *Journal of the American Chemical Society* **140**, 493-498 (2018).
9. Sun, T.M. & Yen, W.T. Kinetics of gold chloride adsorption onto activated carbon. *Minerals Engineering* **6**, 17-29 (1993).
10. Soleimani, M. & Kaghazchi, T. Adsorption of gold ions from industrial wastewater using activated carbon derived from hard shell of apricot stones – An agricultural waste. *Bioresource Technology* **99**, 5374-5383 (2008).
11. Lam, K.F., Yeung, K.L. & McKay, G. An Investigation of Gold Adsorption from a Binary Mixture with Selective Mesoporous Silica Adsorbents. *The Journal of Physical Chemistry B* **110**, 2187-2194 (2006).
12. Nibou, D., Mekatel, H., Amokrane, S., Barkat, M. & Trari, M. Adsorption of $Zn^{2+}$ ions onto NaA and NaX zeolites: Kinetic, equilibrium and thermodynamic studies. *Journal of Hazardous Materials* **173**, 637-646 (2010).
13. Wang, J. et al. Exploration of the adsorption performance and mechanism of zeolitic imidazolate framework-8@graphene oxide for Pb(II) and 1-naphthylamine from aqueous solution. *Journal of Colloid and Interface Science* **542**, 410-420 (2019).
14. Peng, Y. et al. Laser Assisted Solution Synthesis of High Performance Graphene Supported Electrocatalysts. *Advanced Functional Materials* **30**, 2001756 (2020).
15. Wang, R. et al. Interfacial Coordinational Bond Triggered Photoreduction Membrane for Continuous Light-Driven Precious Metals Recovery. *Nano Letters* **23**, 2219-2227 (2023).

16. Zhao, M., Huang, Z., Wang, S. & Zhang, L. Ultrahigh efficient and selective adsorption of Au(III) from water by novel Chitosan-coated $MoS_2$ biosorbents: Performance and mechanisms. *Chemical Engineering Journal* **401**, 126006 (2020).
17. Xue, T. et al. A customized MOF-polymer composite for rapid gold extraction from water matrices. *Science Advances* **9**, eadg4923. (2023).
18. Yu, C.-X. et al. Fabrication of Carboxylate-Functionalized 2D MOF Nanosheet with Caged Cavity for Efficient and Selective Extraction of Uranium from Aqueous Solution. *Small* **20**, 2308910 (2024).
19. Zhang, M. et al. Immobilization of ionic liquids onto chloromethylated polystyrene as a strategy for highly efficient and selective recovery of Au(III) from gold slag and PCBs. *Separation and Purification Technology* **337**, 126479 (2024).
20. Bakhromi, D., Safarmamadzoda, S.M., Fritskii, I.O. & Muborakkadamov, D.A. Complex Formation of $H[AuCl_4]$ with 2-Methylimidazole. *Russian Journal of Inorganic Chemistry* **66**, 820-826 (2021).
21. Mei, D. & Yan, B. A 2D Acridine-Based Covalent Organic Framework for Selective Detection and Efficient Extraction of Gold from Complex Aqueous-Based Matrices. *Angewandte Chemie International Edition* **63**, e202402205 (2024).
22. Li, F. et al. Highly efficient and selective extraction of gold by reduced graphene oxide. *Nature Communications* **13**, 4472 (2022).
23. Li, X. et al. Porous organic polycarbene nanotrap for efficient and selective gold stripping from electronic waste. *Nature Communications* **14**, 263 (2023).
24. Qiu, J. et al. Porous Covalent Organic Framework Based Hydrogen-Bond Nanotrap for the Precise Recognition and Separation of Gold. *Angewandte Chemie International Edition* **62**, e202300459 (2023).
25. Hong, Y. et al. Precious metal recovery from electronic waste by a porous porphyrin polymer. *Proceedings of the National Academy of Sciences* **117**, 16174-16180 (2020).
26. Sun, D.T., Gasilova, N., Yang, S., Oveisi, E. & Queen, W.L. Rapid, Selective Extraction of Trace Amounts of Gold from Complex Water Mixtures with a Metal–Organic Framework (MOF)/Polymer Composite. *Journal of the American Chemical Society* **140**, 16697-16703 (2018).
27. Luo, J. et al. Selective and rapid extraction of trace amount of gold from complex liquids with silver(I)-organic frameworks. *Nature Communications* **13**, 7771 (2022).
28. Kong, H.-Y., Tao, Y., Ding, X. & Han, B.-H. Efficient gold recovery from waste electronic and electric equipment by amino-modified covalent triazine frameworks. *Chemical Engineering Journal* **463**, 142393 (2023).
29. Jung, Y. et al. Nitrogen-Doped Titanium Carbide ($Ti_3C_2T_x$) MXene Nanosheet Stack For Long-Term Stability and Efficacy in Au and Ag Recovery. *Small* **19**, 2305247 (2023).
30. Wang, R. et al. Interfacial Coordination Bonding-Assisted Redox Mechanism-Driven Highly Selective Precious Metal Recovery on Covalent-Functionalized Ultrathin 1T-$MoS_2$. *ACS Applied Materials & Interfaces* **15**, 9331-9340 (2023).
31. Yang, K. et al. Graphene/chitosan nanoreactors for ultrafast and precise recovery and catalytic conversion of gold from electronic waste. *Proceedings of the National Academy of Sciences* **121**, e2414449121 (2024).

32. Shin, S.S. et al. Efficient recovery and recycling/upcycling of precious metals using hydrazide-functionalized star-shaped polymers. *Nature Communications* **15**, 3889 (2024).
33. Liu, F., You, S., Wang, Z. & Liu, Y. Redox-Active Nanohybrid Filter for Selective Recovery of Gold from Water. *ACS ES&T Engineering* **1**, 1342-1350 (2021).
34. Song, K.S. et al. Porous polyisothiocyanurates for selective palladium recovery and heterogeneous catalysis. *Chem* **8**, 2043-2059 (2022).
35. Aguila, B. et al. A Porous Organic Polymer Nanotrap for Efficient Extraction of Palladium. *Angewandte Chemie International Edition* **59**, 19618-19622 (2020).
36. Mu, W. et al. Removal of radioactive palladium based on novel 2D titanium carbides. *Chemical Engineering Journal* **358**, 283-290 (2019).
37. Bai, Y. et al. Precise recognition of palladium through interlaminar chelation in a covalent organic framework. *Chem* **8**, 1442-1459 (2022).
38. Lin, S. et al. Effective adsorption of Pd(II), Pt(IV) and Au(III) by Zr(IV)-based metal–organic frameworks from strongly acidic solutions. *Journal of Materials Chemistry A* **5**, 13557-13564 (2017).
39. Liu, Y. et al. Super-Stable, Highly Efficient, and Recyclable Fibrous Metal–Organic Framework Membranes for Precious Metal Recovery from Strong Acidic Solutions. *Small* **15**, 1805242 (2019).
40. Xiong, J. et al. Cost-effective and high-performance biguanide-incorporated ionic porous organic polymer for selective recovery of Pd(II) and Pt(IV) from metallurgical wastewater. *Chemical Engineering Journal* **488**, 150772 (2024).
41. He, L. et al. Synergy of first- and second-sphere interactions in a covalent organic framework boosts highly selective platinum uptake. *Science China Chemistry* **66**, 783-790 (2023).
42. Fujiwara, K., Ramesh, A., Maki, T., Hasegawa, H. & Ueda, K. Adsorption of platinum (IV), palladium (II) and gold (III) from aqueous solutions onto l-lysine modified crosslinked chitosan resin. *Journal of Hazardous Materials* **146**, 39-50 (2007).
43. Chen, Z., Chan, A.K.-W., Wong, V.C.-H. & Yam, V.W.-W. A Supramolecular Strategy toward an Efficient and Selective Capture of Platinum(II) Complexes. *Journal of the American Chemical Society* **141**, 11204-11211 (2019).
44. Shi, J. et al. Porous Polypyrrolidines for Highly Efficient Recovery of Precious Metals through Reductive Adsorption Mechanism. *Advanced Materials* **36**, 2405731 (2024).
45. Qi, H. et al. Graphdiyne Oxides as Excellent Substrate for Electroless Deposition of Pd Clusters with High Catalytic Activity. *Journal of the American Chemical Society* **137**, 5260-5263 (2015).
46. Shi, X. et al. Protruding Pt single-sites on hexagonal $ZnIn_2S_4$ to accelerate photocatalytic hydrogen evolution. *Nature Communications* **13**, 1287 (2022).
47. Trasatti, S. The absolute electrode potential: an explanatory note (Recommendations 1986). *Pure and Applied Chemistry* **58**, 955-966 (1986).
48. Lide, D.R. CRC Handbook of Chemistry and Physics, 87th Edition. (Taylor & Francis, 2006).
49. Xu, W. et al. Ultrathin transition metal oxychalcogenide catalysts for oxygen evolution in acidic media. *Nature Synthesis* **4**, 327-335 (2025).

50. Tan, J. et al. Metal-lattice-heredity synthesis of single-crystalline 2D transition metal oxides. *Matter* **8**, 101873 (2025).
51. Wang, W. et al. Tantalum pentoxide ($Ta_2O_5$ and $Ta_2O_{5-x}$)-based memristor for photonic in-memory computing application. *Nano Energy* **106**, 108072 (2023).
52. Shi, J. et al. Two-dimensional metallic tantalum disulfide as a hydrogen evolution catalyst. *Nature Communications* **8**, 958 (2017).
53. Suoranta, T., Zugazua, O., Niemelä, M. & Perämäki, P. Recovery of palladium, platinum, rhodium and ruthenium from catalyst materials using microwave-assisted leaching and cloud point extraction. *Hydrometallurgy* **154**, 56-62 (2015).
54. Spooren, J. & Abo Atia, T. Combined microwave assisted roasting and leaching to recover platinum group metals from spent automotive catalysts. *Minerals Engineering* **146**, 106153 (2020).
55. Ohta, M. et al. Thermoelectric properties of $Ti_{1+x}S_2$ prepared by $CS_2$ sulfurization. *Acta Materialia* **60**, 7232-7240 (2012).
56. Peydayesh, M. et al. Gold Recovery from E-Waste by Food-Waste Amyloid Aerogels. *Advanced Materials* **36**, 2310642 (2024).
