# Supplementary material for: Water-mediated recycling of gold, palladium and platinum using semimetallic TiS2 and TaS2 nanosheets
Source: Natl Sci Rev. 2025 Nov 20;13(1):nwaf522. doi: 10.1093/nsr/nwaf522 (PMC12796814; doi:10.1093/nsr/nwaf522)
Supplement: nwaf522_Supplemental_Files [file nwaf522_supplemental_files.zip › Teaser text.docx]

Two-dimensional TiS₂ and TaS₂ nanosheets show ultrahigh adsorption capacities to low-concentration Au, Pd, and Pt ions, allowing efficient recovery of precious metals from their waste streams.
